# Nationwide survey of refractory asthma with bronchiectasis by inflammatory subtypes

**DOI:** 10.1186/s12931-022-02289-y

**Published:** 2022-12-20

**Authors:** Natsuko Nomura, Hisako Matsumoto, Akihito Yokoyama, Yoshihiro Nishimura, Koichiro Asano, Akio Niimi, Yuji Tohda, Norihiro Harada, Hiroyuki Nagase, Makoto Nagata, Hiromasa Inoue, Mitsuko Kondo, Takahiko Horiguchi, Nobuaki Miyahara, Nobuyuki Hizawa, Masayuki Hojo, Noboru Hattori, Naozumi Hashimoto, Akira Yamasaki, Toru Kadowaki, Tomoki Kimura, Mari Miki, Hirokazu Taniguchi, Mikio Toyoshima, Tetsuji Kawamura, Osamu Matsuno, Yoko Sato, Hironobu Sunadome, Tadao Nagasaki, Tsuyoshi Oguma, Toyohiro Hirai, the BEXAS study, Hisashi Ohnishi, Kazuyoshi Imaizumi, Masaki Fujita, Takafumi Suda, Yoichi Takaki, Takashi Kijima, Kazunori Tobino, Makoto Hoshino, Shiro Imokawa, Noriya Hiraoka, Takakazu Sugita, Naomi Miho Ikeda, Kayoko Okamura Hisashi Ohnishi, Junko Terada-Hirashima, Sumito Isogai, Kazuyoshi Imaizumi, Ryosuke Hirano, Masaki Fujita, Tomoyuki Fujisawa, Takafumi Suda, Yoichi Takaki, Naoko Higaki, Shintaro Miyamoto, Taku Nakashima, Hiroshi Iwamoto, Koji Mikami, Toshiyuki Minami, Ryo Takahashi, Takashi Kijima, Kazunori Tobino, Makoto Hoshino, Shiro Imokawa, Taisuke Tsuji, Noriya Hiraoka, Tatsuyoshi Ikeue, Takakazu Sugita, Naomi Kunichika, Shinya Tomari, Yasumi Okochi, Naoko Mato, Koichi Hagiwara, Kunio Dobashi, Yasuyuki Taooka, Kentaro Machida, Takae Tanosaki, Katsunori Masaki, Koichi Fukunaga, Akiko Sano, Takashi Iwanaga, Yuji Higashimoto, Masataka Matsumoto, Kiyonobu Takatsuki, Kazuma Nagata, Ryo Tachikawa, Keisuke Tomii, Masahiro Kaneko, Hiromi Tomioka, Tatsuya Nagano, Mayuka Yamane, Chieko Yoshida, Takuro Sakagami, Yurie Seto, Yoshiko Kaneko, Koichi Takayama, Satoru Terada, Kenta Nishi, Tomoko Tajiri, Saya Nakamura, Keiko Wakahara, Takefumi Ito, Takako Nakano, Takafumi Yamashita, Shohei Takata, Yoshihiro Seri, Yasuyuki Mizumori, Hiroaki Tsukamoto, Ryogo Kagami, Yasuharu Nakahara, Yukio Ishii, Toshiyuki Kita, Kouko Hidaka, Masayoshi Minakuchi, Tomomasa Tsuboi, Shinji Tamaki, Takanori Matsuki, Hiroshi Kida, Katsuyuki Tomita, Takashi Abe, Joe Shindoh, Akihiko Taniguchi, Masato Azuma, Mikio Kataoka, Haruhiko Ogawa, Takeshi Matsumoto, Kensaku Aihara, Kazuyuki Nakagome, Satsuki Miyajima, Kentaro Hashimoto, Tetsuhiro Shiota, Masafumi Yamaguchi, Yasutaka Nakano, Kojiro Otsuka, Masanori Yasuo, Masayuki Hanaoka, Takashi Yamada, Toshihiro Shirai, Yoshinobu Iwasaki, Masamichi Mineshita, Takahiro Tsuburai, Yuko Komase, Hidefumi Koh, Koichi Hasegawa, Hideo Kita, Koji Murakami, Hisatoshi Sugiura, Masakazu Ichinose, Tomoko Kutsuzawa, Tsuyoshi Oguma, Jun Tanaka, Yuta Kono, Shinji Abe, Morio Nakamura, Mami Orimo, Etsuko Tagaya, Toshiaki Matsuda, Tomoya Harada, Hiroaki Iijima, Hiroki Kawabata, Kazuhiro Yatera, Hironori Masuko, Yuko Morishima, Masanori Nakanishi, Nobuyuki Yamamoto, Sumito Inoue, Kazuki Hamada, Yoshikazu Yamaji, Tsunahiko Hirano, Kazuto Matsunaga

**Affiliations:** 1 Department of Respiratory Medicine, Kyoto University Graduate School of Medicine, Kyoto, Japan; 2 Department of Respiratory Medicine and Allergology, Kindai University Faculty of Medicine, 377-2, Ohno-Higashi, Osakasayama, Osaka Japan; 3 Department of Respiratory Medicine and Allergology, Kochi Medical School, Kochi University, Kochi, Japan; 4 Division of Respiratory Medicine, Department of Internal Medicine, Kobe University Graduate School of Medicine, Kobe, Japan; 5 Division of Pulmonary Medicine, Department of Medicine, Tokai University School of Medicine, Kanagawa, Japan; 6 Department of Respiratory Medicine, Allergy and Clinical Immunology, Nagoya City University Graduate School of Medical Sciences, Nagoya, Japan; 7 Department of Respiratory Medicine, Juntendo University Faculty of Medicine and Graduate School of Medicine, Tokyo, Japan; 8 Department of Respiratory Medicine and Allergology, Department of Medicine, Teikyo University School of Medicine, Tokyo, Japan; 9 Department of Respiratory Medicine, Saitama Medical University, Saitama, Japan; 10 Department of Pulmonary Medicine, Graduate School of Medical and Dental Sciences, Kagoshima University, Kagoshima, Japan; 11 Department of Respiratory Medicine, Tokyo Women’s Medical University, Tokyo, Japan; 12 Department of Respiratory Medicine, Toyota Regional Medical Center, Toyota, Japan; 13 Department of Medical Technology, Okayama University Graduate School of Health Sciences, Okayama, Japan; 14 Department of Pulmonary Medicine, Faculty of Medicine, University of Tsukuba, Tsukuba, Japan; 15 Department of Respiratory Medicine, Center Hospital of the National Center for Global Health and Medicine, Tokyo, Japan; 16 Department of Molecular and Internal Medicine, Graduate School of Biomedical and Health Sciences, Hiroshima University, Hiroshima, Japan; 17 Department of Respiratory Medicine, Nagoya University Graduate School of Medicine, Nagoya, Japan; 18 Division of Respiratory Medicine and Rheumatology, Department of Multidisciplinary Internal Medicine, School of Medicine, Faculty of Medicine, Tottori University, Tottori, Japan; 19 Department of Pulmonary Medicine, National Hospital Organization Matsue Medical Center, Matsue, Japan; 20 Department of Respiratory Medicine and Allergy, Tosei General Hospital, Aichi, Japan; 21 Department of Respiratory Medicine, National Hospital Organization Toneyama Medical Center, Osaka, Japan; 22 Department of Respiratory Medicine, Toyama Prefectural Central Hospital, Toyama, Japan; 23 Department of Respiratory Medicine, Hamamatsu Rosai Hospital, Hamamatsu, Japan; 24 Department of Respiratory Medicine, National Hospital Organization Himeji Medical Center, Himeji, Japan; 25 Department of Allergy and Rheumatoid disease, Osaka Habikino Medical Center, Osaka, Japan; 26 Department of Respiratory Medicine, Yuuai Medical Center, Okinawa, Japan; 27 Department of Respiratory Care and Sleep Control Medicine, Kyoto University Graduate School of Medicine, Kyoto, Japan

**Keywords:** Asthma, Blood eosinophil counts, Bronchiectasis, Bronchiolitis, FeNO

## Abstract

**Rationale:**

Bronchiectasis and bronchiolitis are differential diagnoses of asthma; moreover, they are factors associated with worse asthma control.

**Objective:**

We determined clinical courses of bronchiectasis/bronchiolitis-complicated asthma by inflammatory subtypes as well as factors affecting them.

**Methods:**

We conducted a survey of refractory asthma with non-cystic fibrosis bronchiectasis/bronchiolitis in Japan. Cases were classified into three groups, based on the latest fractional exhaled NO (FeNO) level (32 ppb for the threshold) and blood eosinophil counts (320/µL for the threshold): high (type 2-high) or low (type 2-low) FeNO and eosinophil and high FeNO or eosinophil (type 2-intermediate). Clinical courses in groups and factors affecting them were analysed.

**Results:**

In total, 216 cases from 81 facilities were reported, and 142 were stratified: 34, 40 and 68 into the type 2-high, -intermediate and -low groups, respectively. The frequency of bronchopneumonia and exacerbations requiring antibiotics and gram-negative bacteria detection rates were highest in the type 2-low group. Eighty-seven cases had paired latest and oldest available data of FeNO and eosinophil counts; they were analysed for inflammatory transition patterns. Among former type 2-high and -intermediate groups, 32% had recently transitioned to the -low group, to which relatively low FeNO in the past and oral corticosteroid use contributed. Lastly, in cases treated with moderate to high doses of inhaled corticosteroids, the frequencies of exacerbations requiring antibiotics were found to be higher in cases with more severe airway lesions and lower FeNO.

**Conclusions:**

Bronchiectasis/bronchiolitis-complicated refractory asthma is heterogeneous. In patients with sputum symptoms and low FeNO, airway colonisation of pathogenic bacteria and infectious episodes are common; thus, corticosteroids should be carefully used.

**Supplementary Information:**

The online version contains supplementary material available at 10.1186/s12931-022-02289-y.

## Introduction

Bronchiectasis is known to be an important comorbidity and differential diagnosis of severe asthma [1, 2]. Previous studies have shown that patients with asthma complicated with bronchiectasis have refractory disease with frequent exacerbations [3], antibiotic use and bronchopneumonia [4]. However, there is no appropriate guide for the management of bronchiectasis-complicated refractory asthma, which may arise from the heterogeneous nature of this condition.

Asthma is typically characterised by eosinophilic, type 2-high inflammation and bronchiectasis in asthma has been commonly associated with allergic bronchopulmonary aspergillosis (ABPA), i.e., eosinophilic bronchiectasis. Even in patients with asthma complicated with bronchiectasis other than ABPA, average blood eosinophil counts are not decreased [1, 5]. Furthermore, studies on bronchiectasis without asthma or ABPA have shown that eosinophilic predominant phenotypes represented approximately 20% of patients with bronchiectasis [6, 7]. Meanwhile, bronchiectasis has been typically characterised by recurrent airway infection and neutrophilic airway inflammation. Low fractional exhaled nitric oxide (FeNO) levels have been described as markers of comorbid bronchiectasis in asthma [4] and infectious exacerbations in patients treated with anti-interleukin (IL)-5 antibody for severe asthma [8]. Collectively, patients with asthma with comorbid bronchiectasis are heterogeneous, including type 2-high and type 2-low phenotypes. However, their characteristics and clinical courses according to inflammatory phenotypes remain unknown.

Furthermore, cases with bronchiectasis are often accompanied by computed tomography (CT) findings suggestive of chronic infectious bronchiolitis [9]. However, the effects of asthma with comorbid bronchiolitis have rarely been discussed, except for eosinophilic bronchiolitis [10]. Thus, the purpose of this nationwide study was to characterise patients with asthma complicated with bronchiectasis/bronchiolitis, clarify their clinical courses by stratifying them through FeNO and blood eosinophil counts, and examine the roles of these two markers in patient management.

## Methods

### Study design and study population

The BEXAS (bronchiectasis and asthma) study was a nationwide survey conducted at accredited and affiliated facilities of the Japanese Respiratory Society and Japanese Society of Allergology. Patients with refractory asthma complicated by bronchiectasis or bronchiolitis, or both with a history of visits between January 2015 and September 2019 were included. The included cases were resistant to standard management regardless of the doses of inhaled corticosteroid (ICS), and had sputum symptoms. Bronchiectasis was defined as an enlarged bronchoarterial ratio of > 1.1 or lack of tapering of an airway toward the periphery. Detailed assessment of bronchiectasis is shown in Additional file 1. Traction bronchiectasis due to fibrotic interstitial pneumonia, non-tuberculous mycobacterial diseases, cystic fibrosis and acute bronchiolitis cases were excluded.

### Questionnaires

The survey asked attending physicians the following information: basic demographic data, medical history, timing and basis of the asthma diagnosis, timing of the diagnosis and morphological and inflammatory patterns of airway lesions, i.e., bronchiectasis and bronchiolitis, the latest and former treatments and their effectiveness, latest and oldest laboratory data in 5 years, latest and oldest (or at the diagnosis) radiological data, frequencies of exacerbations requiring systemic corticosteroids and antibiotics in the latest 2 years and frequencies of bronchopneumonia and hospital admission due to exacerbations in the latest 5 years. Long-term oral corticosteroid (OCS) use was defined as regular current or past OCS use. Cases with ABPA were excluded from this analysis, as it was considered a separate clinical entity based on its response to established treatments. This study was approved by the Kyoto University Medical Ethics Committee (R2168).

### Statistical analysis

Analyses were performed using JMP version 15. Two or more groups were compared using the χ^2^ test, Fisher’s exact test, Wilcoxon rank-sum test and Kruskal–Wallis test, where deemed appropriate. Multiple comparison tests were performed using Steel–Dwass test. The Wilcoxon signed-rank test was used to compare matched samples. Details are shown in the Additional file 1. A p-value of < 0.05 was considered significant. Data are shown as means (SD).

## Results

### Patient characteristics and stratification by type 2 inflammation

In total, 81 facilities responded, wherein 216 cases were returned, and 35.2% were males and the mean (SD) age was 64.7 (14.6) years. Total of 59% of the patients had severe asthma (details are shown in the Additional file). The mean period from asthma diagnosis to the diagnosis of bronchiectasis/bronchiolitis was 14.4 (17.9) years (n = 180), and the means of current blood eosinophil counts and FeNO levels were 378 (515)/μL (n = 198) and 40 (45) ppb (n = 154), respectively.

**Fig. 1:**
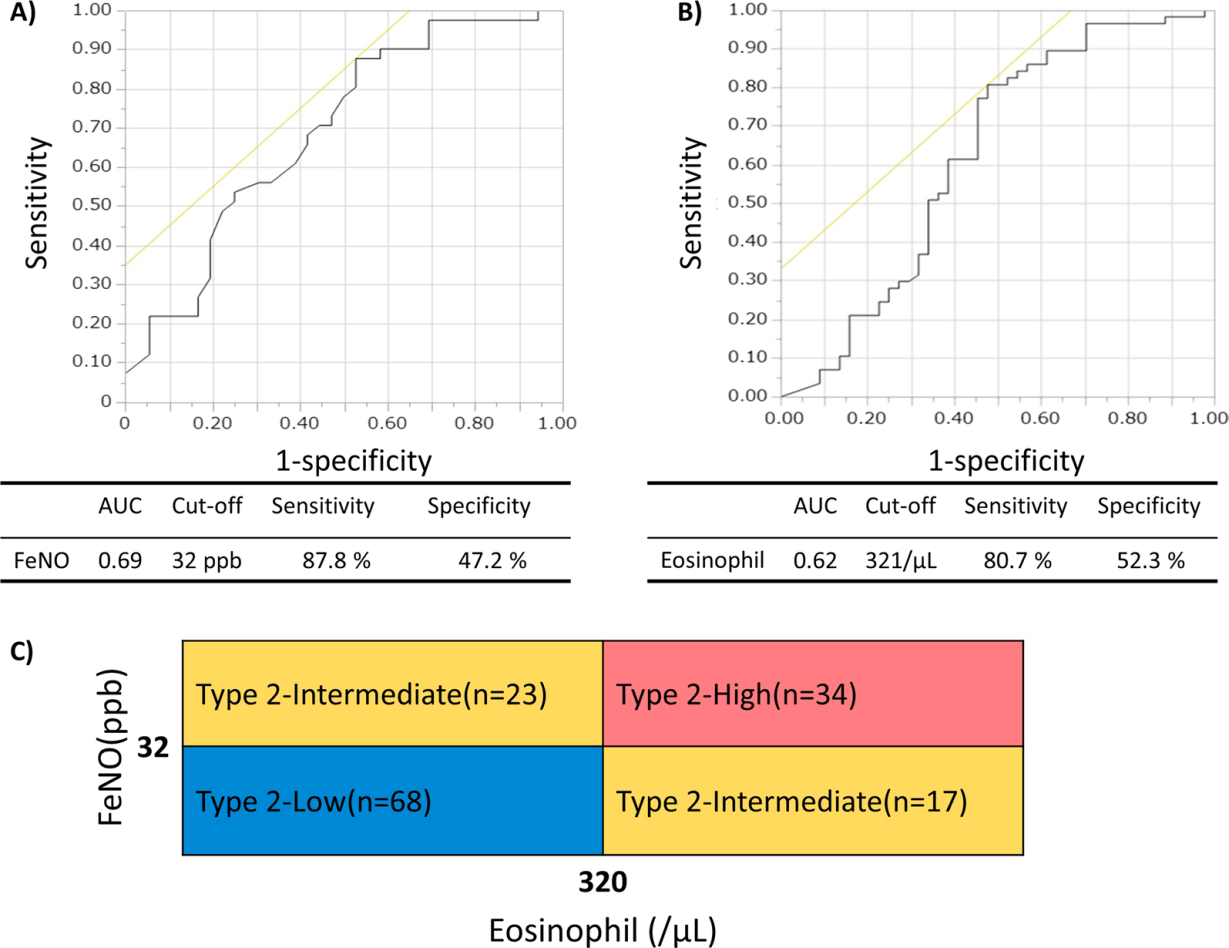
Receiver operating characteristic (ROC) curve of the current levels of **A** exhaled nitric oxide (FeNO) and **B** blood eosinophil counts that reflected the presence of bronchopneumonia in the last 5 years. **C** definition of inflammatory type stratified by FeNO and blood eosinophil counts

First, current FeNO and blood eosinophil levels that reflected the presence of bronchopneumonia in the last 5 years were determined using a receiver operating characteristic (ROC) curve analysis (Fig. 1). The best thresholds were set at an FeNO level of 32 ppb and blood eosinophil count of 321/μL. Using FeNO level of 32 ppb and blood eosinophil count of 320/μL as thresholds, cases with current data on the two type 2-markers were classified into the following three groups: FeNO and eosinophil high (type 2-high, n = 34), FeNO or eosinophil high (type 2-intermediate, n = 40) and FeNO and eosinophil low (type 2-low, n = 68) groups (Fig. 1). Details on the 142 stratified cases and the remaining 74 cases are presented in Additional file 1: Results and Table S1.

### Characteristics and current conditions of the three inflammatory groups

**Fig. 2:**
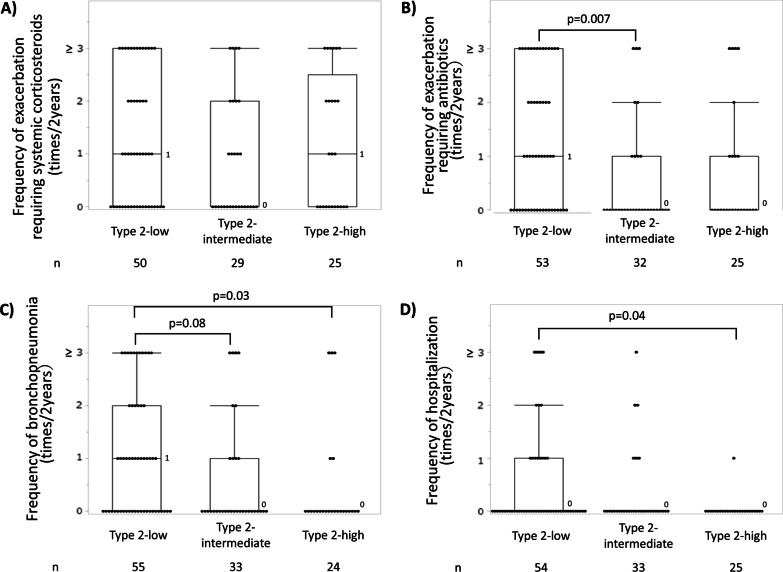
Frequencies of **A** exacerbation requiring systemic corticosteroids (p = 0.30 among the three inflammatory groups by the Kruskal–Wallis test), **B** exacerbation requiring antibiotics (p = 0.005 among the three groups, p = 0.007 for type 2-low vs type 2-intermedate), **C** bronchopneumonia (p = 0.01 among the three groups, p = 0.03 for type 2-low vs type 2-high), and **D** hospitalisation (p = 0.04 among the three groups, p = 0.04 for type 2-low vs type 2-high), in the last 2 years. Boxes and bars indicate upper, lower, and median quartiles

Among the type 2-high, -intermediate and -low groups, no significant differences were noted in terms of age, sex, age at asthma diagnosis, or period from asthma diagnosis to bronchiectasis/bronchiolitis diagnosis (Table 1). The type 2-low group showed the lowest serum total IgE levels and highest serum C-reactive protein levels, and patients’ sputum culture revealed gram-negative bacteria (GNB) and *Pseudomonas aeruginosa* (*P. aeruginosa*) most frequently among the three inflammatory groups (Table 1). As expected, the type 2-low group had infectious episodes most frequently (Fig. 2), but the frequencies of asthma exacerbation requiring systemic corticosteroids did not differ among the three groups. The frequencies of other comorbidities did not differ among the groups (Additional file 1: Table S2). Anti-type 2 biologics or regular OCS (≥ 5 mg/day) was administered to 56 cases, and the frequency of administration did not differ among the three groups. The major features of the inflammatory groups did not change when the study was limited to subjects not receiving these medications (Additional file 1: Results and Fig. S1). Impression of the attending physicians is described in the Additional file 1: Results.

**Table 1:** Patient characteristics and current conditions according to inflammatory types

Current inflammatory type	N^‡^	Type 2-low N = 68	Type 2-intermediate N = 40	Type 2-high N = 34	p-value
Age, years	142	62.7 ± 16.5	62.9 ± 13.7	64.7 ± 12.3	0.89
Sex, male, n (%)	142	18 (26.5)	12 (30.0)	15 (44.1)	0.19
Body mass index, kg/m^2^	121	22.8 ± 3.6	22.5 ± 5.0	23.2 ± 4.5	0.43
Follow-up period, years	136	7.9 ± 5.8^§^	6.9 ± 5.6	5.3 ± 6.0	0.04
Smoking history, never/past/current, %	140	70/28/2	63/30/8	70/24/6	0.58
Age at diagnosis of asthma, years	135	40.4 ± 22.4	40.5 ± 21.8	48.4 ± 19.1	0.15
Age at diagnosis of bronchiectasis/bronchiolitis, years	122	55.8 ± 17.6	59.3 ± 15.9	60.5 ± 15.2	0.48
Period from asthma diagnosis to bronchiectasis/ bronchiolitis diagnosis, years	117	13.1 ± 15.2	19.8 ± 20.6	12.8 ± 13.6	0.40
ICS dose, μg/day (eq. to fluticasone propionate)	139	613 ± 328^§^	671 ± 364	847 ± 401	0.02
Long-acting β_2_ agonist, n (%)	141	63 (92.7)	37 (92.5)	31 (93.9)	0.97
Long-acting muscarinic antagonist, n (%)	141	42 (61.8)^§^	17 (42.5)	8 (24.2)	0.001
Macrolide therapy, n (%)	141	40 (59.7)^§^	16 (40.0)	10 (29.4)	0.009
Biologics use, n (%)	142	17 (25.0)	11 (27.5)	4 (11.8)	0.22
OCS use, never/past/current, n	137	39/8/19	24/5/9	20/8/5	0.40
OCS dose in long-term OCS* users, < 5 mg/day / ≥ 5 mg/day /missing (eq. to prednisolone), n	54	8/16/3	1/13/0	5/8/0	0.13
Mucolytic agent use, n (%)	140	38 (55.9)^§^	14 (35.9)	8 (24.2)	0.006
Physiotherapy or airway clearance, n (%)	140	2 (2.9)	2 (5.1)	1 (3.0)	0.83
Blood white cell counts, /μL	142	7705 ± 2691	7414 ± 2619	7608 ± 2002	0.69
Neutrophil counts, /μL		5407 ± 2611	4783 ± 2310	4415 ± 1831	0.15
Eosinophil counts, /μL		121 ± 95^§¶^	467 ± 775^§^	870 ± 480	< 0.0001
Serum total IgE, IU/mL	88	275 ± 335^§^	446 ± 607	573 ± 683	0.02
Serum C-reactive protein, mg/dL	116	1.6 ± 3.3^§^	0.9 ± 2.1	0.2 ± 0.2	0.005
Exhaled nitrate oxide, ppb	142	15.9 ± 8.0^§¶^	50.5 ± 41.3^§^	82.4 ± 63.0	< 0.0001
%FEV_1_, %	126	78.7 ± 32.5	88.4 ± 22.5	87.5 ± 31.9	0.045
FEV_1_/FVC, %	126	66.9 ± 16.0	69.8 ± 12.8	64.9 ± 13.2	0.39
Findings of computed tomography					
Bronchiectasis/bronchiolitis/both, %		14/28/58	28/24/48	21/33/46	0.54
Modified Reiff score	134	3.4 ± 3.6	3.1 ± 3.3	1.9 ± 2.5	0.13
Number of lobes affected by bronchiolitis	124	3.4 ± 2.4	2.3 ± 2.0	2.7 ± 2.2	0.043
Sputum *S. pneumonia* ( +), n (%)	102	6 (11.3)	2 (6.9)	4 (20.0)	0.37
Gram-negative bacteria ( +), n (%)	102	32 (60.4)^§¶^	7 (24.1)	4 (20.0)	0.0005
*P. aeruginosa* ( +), n (%)	102	21 (39.6)^§¶^	3 (10.3)	2 (10.0)	0.003
Presence of exacerbations requiring systemic corticosteroid in the last 2 years^†^, n (%)	104	31 (62.0)	13 (44.8)	14 (56.0)	0.33
Presence of exacerbations requiring antibiotics in the last 2 years^†^, n (%)	110	33 (62.3)	9 (28.1)	9 (36.0)	0.005
Presence of bronchopneumonia in the last 2 years^†^, n (%)	112	31 (56.4)^§¶^	10 (30.3)	5 (20.8)	0.004
Presence of hospitalisation for exacerbation in the last 2 years^†^, n (%)	112	15 (27.8)^§^	6 (18.2)	1 (4.0)	0.045

Data are presented as means ± SD. Most recent data are presented, if not otherwise stated. *FEV*_*1*_ forced expiratory volume in 1 s, *FVC* forced vital capacity, *ICS* inhaled corticosteroid, *OCS* oral corticosteroid. *Long-term OCS use was defined as regular current or past OCS use. ^†^Examined in cases who were followed for 2 years or more. ^‡^Number of responded cases for each item, ^§^p < 0.05 vs type 2-high group, ^¶^p < 0.05 vs type 2-intermediate group

Although the composite of the two type 2-markers described the examined cases well, lower FeNO alone also indicated the presence of GNB and *P. aeruginosa* in patient’s sputum and more severe airway lesions. The areas under the curves (AUCs) of ROC curves for predicting sputum GNB and *P. aeruginosa* were 0.74 and 0.74, respectively (Additional file 1: Results and Fig. S2). The AUCs of ROC curves for blood eosinophil counts were 0.54 for GNB and 0.57 for *P. aeruginosa* in the sputum (data not shown). Lower FeNO alone, but not lower blood eosinophil counts, was also associated with the degree of bronchiectasis and bronchiolitis (Additional file 1: Fig. S3).

### Transition pattern of inflammatory types

Subsequently, the transition pattern of inflammatory types was examined in cases that were followed up for ≥ 2 years as well as had paired latest and oldest available data of FeNO and blood eosinophil counts. The mean (SD) period between the latest and oldest data was 3.9 (2.0) years. Former inflammatory types were similarly determined using the thresholds of FeNO (32 ppb) and blood eosinophil (320/μL). Cases who were included in the transition analysis (n = 87) had more items related to asthma diagnosis [3.3 (1.4) vs 2.4 (1.3), p < 0.0001] and less frequently yielded GNB in the past (32% vs 58%, p = 0.004) compared with the remaining cases who were followed for ≥ 2 years but did not have paired data of type 2-markers (n = 90).

**Fig. 3:**
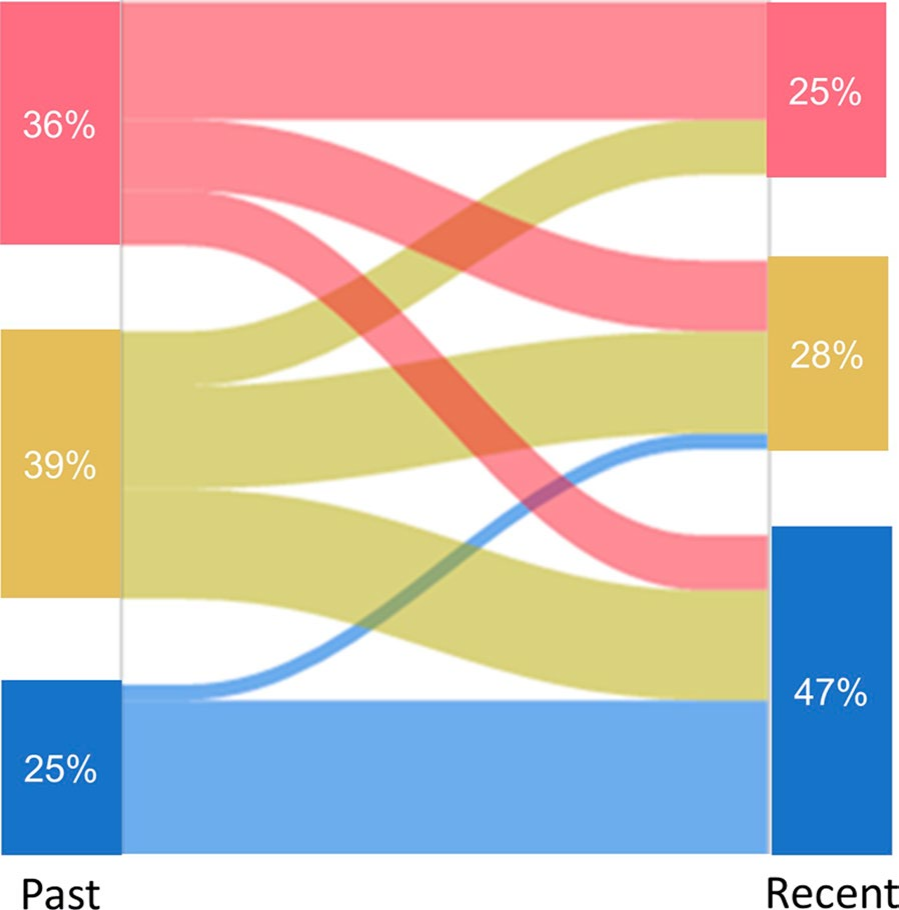
Transition patterns of inflammatory types in cases followed for 2 years or more (n = 87). Red flow indicates former type 2-high group (n = 31); yellow flow, former type 2-intermediate group (n = 34); blue flow, former type 2-low inflammatory group (n = 22)

**Fig. 4:**
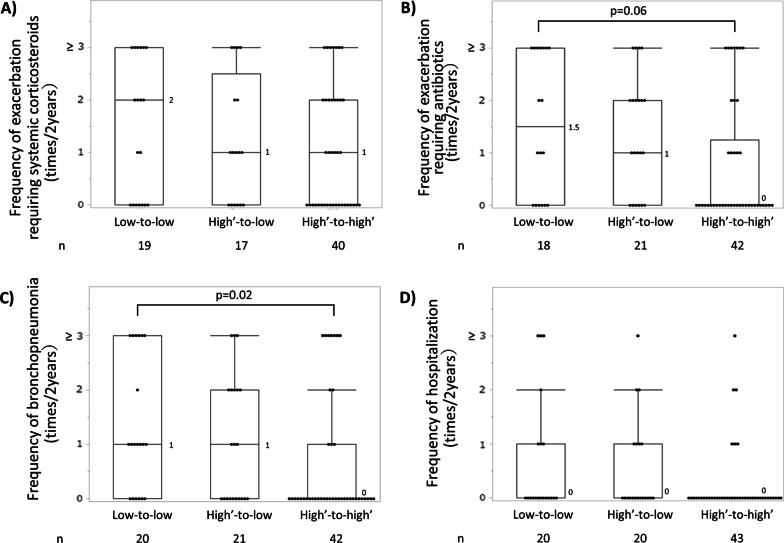
Frequencies of **A** exacerbation requiring systemic corticosteroids (p = 0.51 among the three transition patterns by the Kruskal–Wallis test), **B** exacerbation requiring antibiotics (p = 0.02 among the three patterns and p = 0.06 for low-to-low vs high’-to-high’), **C** bronchopneumonia (p = 0.03 among the three patterns and p = 0.02 for low-to-low vs high’-to-high’), and **D** hospitalisation (p = 0.08), in the last 2 years. Boxes and bars indicate upper, lower, and median quartiles

Figure 3 shows the transition pattern of 87 cases. Among the 22 cases (25%) of the former type 2-low group, 20 remained in the type 2-low group based on the latest available data (defined as low-to-low) and 2 transitioned to the type-2 intermediate group. Among the 65 cases (75%) of former type 2-high and -intermediate groups, 21 transitioned to the current type 2-low group (high’-to-low) and 44 remained in the original category (high’-to-high’); in this transition analysis, the high’ group included type 2-high and -intermediate groups. In the high’-to-high’ group, the frequencies of exacerbations requiring antibiotics and bronchopneumonia in the last 2 years (Fig. 4 and Additional file 1: Fig. S4) were found to be the lowest, whereas the current daily doses of ICS were the highest (Table 2). The three transition groups had exacerbations similarly requiring systemic corticosteroids (63% in the low-to-low group, 65% in the high’-to-low and 53% in high’-to-high’ group, p = 0.60). In the high’-to-high’ group, the ICS dose was significantly increased over time (p = 0.001) (Additional file 1: Fig. S5).

**Table 2:** Patient characteristics according to the transition patterns of inflammatory types

	N^‡^	Low-to-low N = 20	High’-to-low N = 21	High’-to-high’ N = 44	p-value
Age, years	85	62.0 ± 15.2	71.3 ± 11.1	64.6 ± 12.1	0.08
Sex, male, n (%)	85	7 (35.0)	8 (38.1)	16 (36.4)	0.98
Body mass index, kg/m^2^	73	22.9 ± 3.9	22.2 ± 4.2	22.7 ± 4.3	0.90
Follow-up period, years	85	7.8 ± 5.4	9.3 ± 5.3	7.8 ± 6.2	0.29
Smoking history, never/past/current, %	84	60/40/0	71/29/0	61/33/7	0.46
Age at diagnosis of asthma, years	82	37.6 ± 21.8	51.4 ± 21.3	44.3 ± 19.9	0.14
Age at diagnosis of bronchiectasis/bronchiolitis, years	76	56.9 ± 14.7	64.3 ± 11.0	59.3 ± 14.9	0.32
Period from asthma diagnosis to bronchiectasis/bronchiolitis diagnosis, years	74	19.3 ± 19.3^§^	5.9 ± 7.1	15.1 ± 15.8	0.03
Current ICS dose, μg/day (eq. to FP)	83	513 ± 317^¶^	698 ± 303	802 ± 400	0.03
Maximum dose of ICS in the past, μg/day (eq. to FP)	73	653 ± 322	575 ± 438	536 ± 407	0.54
OCS use, never/past/current	83	16/2/2^§^	7/2/11	27/8/8	0.01
OCS dose in long-term OCS* user, < 5 mg/day / ≥ 5 mg/day/missing (eq. to prednisolone), n	33	1/3/0	5/7/1	5/11/0	0.78
Most effective treatment in stable phase^†^ ICS, n (%)	84	5 (26.3)	2 (9.5)^¶^	23 (52.3)	0.002
OCS, n (%)	85	2 (10.0) ^§^	8 (38.1)^¶^	6 (13.6)	0.03
Macrolide therapy, n (%)	85	5 (25.0) ^¶^	4 (19.0)	3 (6.8)	0.12
Most effective treatment on exacerbation^†^ SCS, n (%)	82	7 (35.0)	12 (63.2)	24 (55.8)	0.17
Antibiotics, n (%)	78	6 (31.6) ^¶^	5 (26.3)^¶^	2 (5.0)	0.02
Current data: Blood eosinophil counts, /μL	85	127 ± 98^¶^	144 ± 98^¶^	599 ± 575	< 0.0001
Serum total IgE, IU/mL	53	224 ± 312^¶^	384 ± 386	552 ± 670	0.055
Serum C-reactive protein, mg/dL	69	1.0 ± 2.0	1.0 ± 1.4	0.7 ± 2.1	0.37
Exhaled nitric oxide, ppb	85	15.3 ± 7.1^¶^	18.5 ± 8.1^¶^	71.4 ± 55.3	< 0.0001
Modified Reiff score	82	2.6 ± 3.6	3.9 ± 3.9	2.2 ± 2.3	0.37
Number of lobes affected by bronchiolitis	75	3.6 ± 2.2	3.5 ± 2.6	2.1 ± 2.1	0.02
Gram-negative bacteria ( +) in sputum, n (%)	67	12 (66.7) ^¶^	9 (56.3)^§^	4 (12.1)	0.0001
*P. aeruginosa* ( +) in the latest sputum, n (%)	67	7 (38.9) ^¶^	6 (37.5)^§^	1 (3.0)	0.002
Past data: Blood eosinophil counts, /μL	85	132 ± 85^§¶^	529 ± 346	645 ± 545	< 0.0001
Serum total IgE, IU/mL	57	434 ± 562	372 ± 451	1271 ± 2952	0.10
Serum C-reactive protein, mg/dL	71	1.6 ± 2.0^¶^	0.5 ± 0.5	0.3 ± 0.4	0.006
Exhaled nitric oxide, ppb	85	13.5 ± 6.5^§¶^	37.9 ± 19.5^¶^	75.5 ± 62.3	< 0.0001
Modified Reiff score	82	3.2 ± 3.5	2.8 ± 2.9	1.9 ± 2.3	0.30
Number of lobes affected by bronchiolitis	72	3.7 ± 1.8^¶^	3.0 ± 2.3	2.3 ± 2.0	0.06
Gram-negative bacteria ( +) in sputum, n (%)	71	8 (44.4)	7 (38.9)	8 (22.9)	0.22
*P. aeruginosa* ( +) in sputum, n (%)	71	4 (22.2)	5 (27.8)^¶^	2 (5.7)	0.07
Presence of hospitalisation for exacerbation in the last 2 years, n (%)	83	7 (35.0)	7 (35.0)	6 (14.0)	0.08

Unless otherwise specified, the table presents current data. Data are presented as means ± SD. *FP* fluticasone propionate, *ICS* inhaled corticosteroid, *OCS* oral corticosteroid, *SCS* systemic corticosteroid. *Long-term OCS use was defined as regular current or past OCS use. ^†^Evaluated by attending physicians. ^‡^Number of responses for each item, ^§^p < 0.05 vs High’-to-low group, ^¶^p < 0.05 vs High’-to-high’ group

The high’-to-low group showed an approximately equal number of positive items associated with asthma diagnosis as the high’-to-high’ group (Additional file 1: Table S4) and a *P. aeruginosa* detection rate similar to that of the low-to-low group (Table 2). Furthermore, in the high’-to-low group, the rate of long-term OCS use was the highest, and the period between the diagnosis of asthma and bronchiectasis/bronchiolitis was the shortest, among the three groups.

To identify the factors associated with high’-to-low transition, multivariate logistic regression analysis was performed in patients of former type 2-high or -intermediate groups. Long-term OCS use, lower FeNO in the past, and a shorter period between the diagnosis of asthma and airway lesions were independent contributing factors to the high’-to-low transition (Table 3). Details of airway lesions and their transition patterns are presented in the Additional file 1: Results.

**Table 3:** Factors associated with transition from former type 2-high or -intermediate groups to the current type 2-low group

	Odds (95% confidence interval)	p-value
Age, year	1.08 (0.99–1.16)	0.054
Sex, female	1.21 (0.22–6.55)	0.82
Period between diagnosis of asthma and bronchiectasis/bronchiolitis, year	0.89 (0.81–0.98)	0.02
Long-term OCS use^*^, yes	10.7 (1.45–79.3)	0.02
Exhaled nitric oxide in the past, ppb	0.96 (0.93–0.99)	0.04
Blood eosinophil count in the past, /μL	0.999 (0.997–1.001)	0.32
N = 53	R^2^	0.39

*OCS* oral corticosteroid. *Long-term OCS use was defined as regular current or past OCS use

**Fig. 5:**
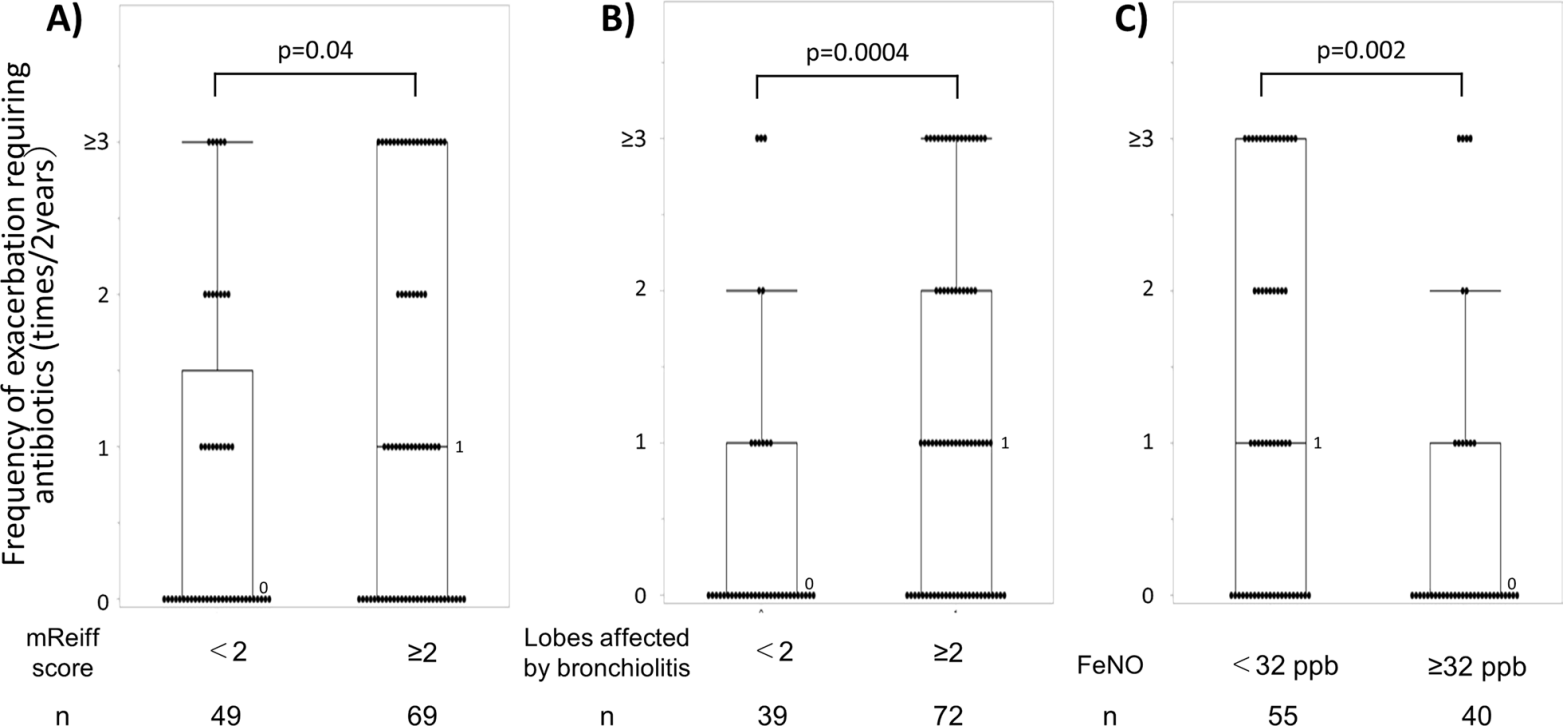
Frequency of exacerbation requiring antibiotics in the last 2 years in cases treated with moderate to high doses of inhaled corticosteroids according to the current degree of **A** bronchiectasis, i.e., modified Reiff (mReiff) score, **B** bronchiolitis, and **C** FeNO level. Boxes and bars indicate upper, lower, and median quartiles

Additionally, among cases treated with moderate to high ICS doses, the frequencies of exacerbations requiring antibiotics in the last 2 years were higher in cases with mReiff scores ≥ 2 and bronchiolitis in ≥ 2 lobes on the latest CT, which was also observed when stratified by a recent FeNO of 32 ppb (Fig. 5). These differences were not observed in cases with zero to low ICS doses.

## Discussion

This is the first nationwide survey clarifying the characteristics and clinical courses of refractory asthma complicated with bronchiectasis/bronchiolitis by stratifying patients according to FeNO and blood eosinophil levels. As expected, the current type 2-low inflammatory group, which accounted for approximately half of the studied cases, was found to have infectious episodes most frequently among the three inflammatory groups, with the detection of GNB and *P. aeruginosa* in the sputum. Meanwhile, the current type 2-high group did not suffer from infectious episodes, despite the presence of bronchiectasis and using the highest ICS dose. However, nearly one-third of cases of former type 2-high or -intermediate groups transitioned to the current type 2-low group (high’-to-low), and this transition was contributed by relatively low FeNO levels in the past and long-term OCS use.

Stratification using FeNO and blood eosinophils provided a clear picture of the characteristics and transition patterns of patients with refractory asthma and bronchiectasis/bronchiolitis. Patients in the type 2-low group had the highest number of infectious episodes and hospitalisations, but they required systemic corticosteroids due to exacerbations similar to type 2-high and -intermediate groups. Importantly, nearly half of the current type 2-low group cases were from former type 2-high or -intermediate groups. This high’-to-low group was indistinguishable from the low-to-low group as per current laboratory data and CT findings, but its blood eosinophil counts in the past were as high as those of the high’-to-high’ group.

Low FeNO levels and blood eosinophil counts may indicate type 2-low inflammation. In this study, FeNO performed better than blood eosinophil counts in the detection of GNB and *P. aeruginosa* in the sputum, reflecting more severe airway lesions. FeNO was shown to be reduced and negatively correlated with the number of lobes affected by bronchiectasis in non-asthmatic patients with *P. aeruginosa* in the sputum [11]. Low FeNO is also suggestive of infectious exacerbations under anti-IL-5 antibody (mepolizumab) treatment in severe asthma [8]. The risk of bronchiectasis comorbidity can be predicted by lower levels of Fe**NO**, a previous history of **P**neumonia, the presence of chronic **E**xpectoration, and the **S**everity of asthma (NOPES score) [4]. For eosinophil counts, a negative correlation between tissue eosinophil counts and the DNA abundance of the phylum *Proteobacteria* in the airways of severe asthma is also shown [12] and eosinophils can activate innate and adaptive immune responses against microbials [13–15], including the eradication of *P. aeruginosa* biofilms through their granules [16]. Nonetheless, the roles of blood eosinophil counts in predicting bacterial colonisation or infectious episodes in clinical settings remain to be unknown. The mechanisms underlying the decrease in FeNO in chronic suppurative conditions are not clearly demonstrated, but are likely due to poor NO diffusion across the viscous and increased airway secretions and the removal of NO by reacting with reactive oxygen species [17]. Decreases in FeNO may reflect the impaired antimicrobial activity in the damaged airways, considering that NO attacks viruses and bacteria to prevent infection [18–20]. Inducible NO synthase is induced by not only type 2-cytokines, but also antimicrobial cytokines, such as tumour necrosis factor-α, interferon-γ and IL-1β [21]. Clinically, it is difficult to determine whether low FeNO reflects optimally treated asthma or bacterial airway colonisation and the development of bronchiectasis/bronchiolitis, but it is better to consider the possibility of bronchiectasis/bronchiolitis when patients with optimally treated asthma only had purulent sputum and showed low FeNO. Further prospective studies are warranted.

The risks associated with long-term OCS use are well-known. As this was a retrospective study over two time points and details of treatments over the two time points were unavailable, the interpretations and conclusions should be made with caution. Nonetheless, the present study showed its potential risk of infection in refractory asthma complicated with bronchiectasis/bronchiolitis, by showing that long-term OCS use was associated with the transition from former type 2-high or -intermediate groups to the current type 2-low group, independent of lower FeNO in the past. Meanwhile, ICS did not affect the above transition. ICS efficiently suppresses airway inflammation, reduces mucus obstruction and protects against bacterial airway invasion [22]. Additionally, unlike chronic obstructive pulmonary disease [23] or bronchiectasis, the risks of pneumonia in ICS for asthma can vary among studies [24–26]. Nonetheless, ICS may be a double-edged sword, particularly at high doses, as is alerted elsewhere [27, 28]. This was likely especially in type 2-low and structurally damaged airways, as seen in the increased exacerbations requiring antibiotics in cases treated with moderate to high ICS doses in this study. Several studies have showed a loss of diversity in microbiota composition [29] and increased pathogenic *Proteobacteria*, particularly *Haemophilus influenzae* and *P. aeruginosa,* in the airways of patients with asthma receiving ICS either with or without OCS compared to those without any corticosteroid use [30, 31]. This may be associated with an increased risk of lower respiratory tract infection in susceptible patients in the later years. Guidelines for bronchiectasis recommend the non-use of ICS, except for in cases of concomitant asthma [32, 33]. However, in patients with asthma complicated by severer bronchiectasis/bronchiolitis with type 2-low inflammation, lower ICS doses may be preferable and physiotherapy, including airway clearance should be implemented, as in bronchiectasis without asthma.

A previous microbiota study on asthma reported that patients with type 2-high asthma had a significantly lower bronchial bacterial burden than those with type 2-low asthma [34]. Consistent with the results of a previous study, the type 2-high group, which represented 24% of the studied population, had least number episodes of infection and GNB and *P. aeruginosa* in sputum despite the use of the highest dose of ICS. Although bronchiolitis pathology was not examined here, centrilobular micronodules on CT in this group may have included eosinophilic bronchiolitis. Neutrophilic and eosinophilic airways may have different microbiome composition [12, 35], which could be explained by the effect of microbial itself and the selective pressure of airway inflammatory types [29]. Further studies on the mechanisms underlying these differences are thus required.

The comorbidity of chronic infectious bronchiolitis in asthma has been rarely discussed; however, the degree of bronchiolitis was associated with infectious episodes in this study. Furthermore, 30% of former bronchiolitis developed into bronchiectasis. The mechanisms underlying this are yet to be determined, but small airway inflammation may cause the dilatation of larger adjacent airways through protease/elastase secretion from neutrophils [36]. Although not focusing on small airways, studies on children showed that 12% of protracted bacterial bronchitis cases progressed to bronchiectasis [37]. Overall, bronchiolitis may be a possible precursor of bronchiectasis. CT findings suggestive of chronic infectious bronchiolitis should be noted in the management of refractory asthma with sputum symptoms and low FeNO.

Diagnosing asthma with bronchiectasis is always a dilemma, but bronchiectasis has often been considered a consequence of severe, uncontrolled asthma, as bronchial dilatation on CT is known to be more prevalent in patients with asthma than in healthy subjects, and the degree of dilatation is associated with asthma severity [38, 39]. According to a study by Mäntyä et al*.*, when asthma and bronchiectasis overlap, the focus should be on the time of diagnosis of each disease; moreover, an earlier diagnosis of asthma may indicate that bronchiectasis is a consequence of asthma [40]. Similar to their findings, wherein the diagnosis of asthma preceded that of bronchiectasis by an average of 18.5 years, in our study, the diagnosis of asthma preceded that of bronchiectasis by 14 years. Additionally, systemic corticosteroid use was similarly required in the low-to-low group, which may also support the presence of asthma in this group.

This study has some limitations. First, the timing, frequencies, types, procedures of examinations and diagnostic and treatment strategies were not standardised among the facilities, due to the multicenter retrospective survey nature of this study. The transition patterns of inflammatory types were determined using two data points. However, the trajectories of blood eosinophil counts from patients with ≥ 3 data points appeared to show believable differences among the three transition patterns (Additional file 1: Fig. S7). In this study, the definition of bronchiectasis did not satisfy the latest recommendation in clinical trials [41]; however, we enroled cases with radiological diagnosis of bronchiectasis and sputum symptoms. In addition, the aetiology of exacerbations may not be sufficiently accurate, but the frequency of exacerbations requiring antibiotics was strongly associated with that of bronchopneumonia (rho = 0.78, p < 0.0001). Lastly the cases stratified by FeNO and blood eosinophil counts were part of the collected cases, but the stratified cases showed more items related to asthma diagnosis (Additional file 1: Table S1) than those which were not stratified. We believe that the stratified cases were a proper population for this analysis of refractory asthma.

In conclusion, this comprehensive nationwide survey suggests that refractory asthma complicated by bronchiectasis/bronchiolitis was heterogeneous. Long-term OCS use may increase the risk of transition from the originally high or intermediate type 2 to type 2-low inflammation with infectious episodes. Low FeNO may serve as a clinical indicator of this transition in patients with sputum symtoms despite optimal asthma treatment. Further prospective studies are needed to confirm the findings.

## Supplementary Information

**Additional file 1: Figure S1. **Transition patterns of inflammatory types in cases who were not receiving anti-type 2 biologics or regular oral corticosteroids (≥5 mg/day) and were followed up for ≥2 years (n = 51). Red flow indicates former type 2-high group (n = 15), yellow flow indicates former type 2-intermediate group (n = 21) and blue flow indicates former type 2-low inflammatory group (n = 15). **Figure S2. **Receiver operating characteristic (ROC) curve for current levels of exhaled nitric oxide (FeNO) that reflected the presence of A) gram-negative bacteria (GNB) and B) *P. aeruginosa* in the sputum. Detection rate of C) GNB and D) *P. aeruginosa* in the sputum according to the FeNO levels, as determined by ROC curve analysis. **Figure S3. **A) Modified Reiff score and B) number of lobes affected by bronchiolitis in low and high exhaled nitric oxide (FeNO) groups. Recent indices were analysed. Boxes and bars indicate upper, lower, and median quartiles. **Figure S4.** Patterns of cases with exacerbations requiring systemic corticosteroids (SCS) and antibiotics and bronchopneumonia, according to the transition patterns of inflammatory groups (p = 0.056 among the three groups). Red bar indicates cases with three types of episodes in the last 2 years, i.e., exacerbations requiring SCS and antibiotics, and bronchopneumonia; orange bar, exacerbations requiring SCS and antibiotics; yellow bar, exacerbations requiring SCS only: blue bar, bronchopneumonia and exacerbations requiring antibiotics; purple bar, bronchopneumonia only; green bar, exacerbations requiring antibiotics only. Complete answers were missing from two cases in the low-to-low group, four in the high’-to-low group, and eight in the high’-to-high’ group. Ratios of cases with exacerbation requiring antibiotics (p = 0.01) and bronchopneumonia (p = 0.006) were significantly different among the three groups. **Figure S5. **Changes in terms of A) inhaled corticosteroid (ICS) doses (equivalent to fluticasone propionate), B) modified Reiff scores, C) number of lobes affected by bronchiolitis, D) detection rates of gram-negative bacteria (GNB) in sputum and E) detection rates of *P. aeruginosa* in sputum over time. Blue line indicates low-to-low group, yellow line indicates high’-to-low group and pink line indicates high’-to-high’ group. The numbers of cases in the low-to-low, high’-to-low, and high’-to-high’ groups were 17/19/35 for A), 19/21/40 for B); 15/21/34 for C); 17/15/32 for D) and 17/15/32 for E). **Figure S6.** Transition patterns of airway lesions, i.e., bronchiectasis, bronchiolitis, and both, in cases followed for 2 years or more. A) In all cases regardless of type 2 inflammation level, purple flow indicates former bronchiectasis only (n = 17); orange flow, former bronchiolitis only (n = 36); green flow, bronchiectasis and bronchiolitis in the past (n = 63); B) current type 2-high group (n = 14), C) current type 2-intermediate group (n = 17) and D) current type 2-low group (n = 41). **Figure S7.** Trajectories of blood eosinophil counts of patients with ≥3 data points allocated to A) high’-to-high’, B) high’-to-low and C) low-to-low groups. Patients represented by grey and brown lines in A), who received oral corticosteroids or anti-type 2 biologics, or both, had elevated exhaled nitric oxide (>100 ppb), and they were allocated to the high’-to-high group. **Table S1.** Characteristics of patients who were and were not stratified. **Table S2.** Features related to asthma and comorbidities according to the current inflammatory types. **Table S3.** Most effective treatment evaluated by attending physicians. **Table S4**. Features related to asthma according to the transition patterns of inflammatory types.

## Data Availability

Not applicable.
