# Supplementary material for: Nationwide survey of refractory asthma with bronchiectasis by inflammatory subtypes
Source: Respir Res. 2022 Dec 20;23:365. doi: 10.1186/s12931-022-02289-y (PMC9763800; doi:10.1186/s12931-022-02289-y)
Supplement: Supplementary file 1 — Additional file 1: Figure S1. Transition patterns of inflammatory types in cases who were not receiving anti-type 2 biologics or regular oral corticosteroids (≥5 mg/day) and were followed up for ≥2 years (n = 51). Red flow indicates former type 2-high group (n = 15), yellow flow indicates former type 2-intermediate group (n = 21) and blue flow indicates former type 2-low inflammatory group (n = 15). Figure S2. Receiver operating characteristic (ROC) curve for current levels of exhaled nitric oxide (FeNO) that reflected the presence of A) gram-negative bacteria (GNB) and B) P. aeruginosa in the sputum. Detection rate of C) GNB and D) P. aeruginosa in the sputum according to the FeNO levels, as determined by ROC curve analysis. Figure S3. A) Modified Reiff score and B) number of lobes affected by bronchiolitis in low and high exhaled nitric oxide (FeNO) groups. Recent indices were analysed. Boxes and bars indicate upper, lower, and median quartiles. Figure S4. Patterns of cases with exacerbations requiring systemic corticosteroids (SCS) and antibiotics and bronchopneumonia, according to the transition patterns of inflammatory groups (p = 0.056 among the three groups). Red bar indicates cases with three types of episodes in the last 2 years, i.e., exacerbations requiring SCS and antibiotics, and bronchopneumonia; orange bar, exacerbations requiring SCS and antibiotics; yellow bar, exacerbations requiring SCS only: blue bar, bronchopneumonia and exacerbations requiring antibiotics; purple bar, bronchopneumonia only; green bar, exacerbations requiring antibiotics only. Complete answers were missing from two cases in the low-to-low group, four in the high’-to-low group, and eight in the high’-to-high’ group. Ratios of cases with exacerbation requiring antibiotics (p = 0.01) and bronchopneumonia (p = 0.006) were significantly different among the three groups. Figure S5. Changes in terms of A) inhaled corticosteroid (ICS) doses (equivalent to fluticasone propionate), B) mod [file 12931_2022_2289_MOESM1_ESM.docx]

**Additional file 1**

**Nationwide Survey of Refractory Asthma with Bronchiectasis by Inflammatory Subtypes**

Natsuko Nomura, Hisako Matsumoto, Akihito Yokoyama, Yoshihiro Nishimura, Koichiro Asano, Akio Niimi, Yuji Tohda, Norihiro Harada,　Hiroyuki Nagase, Makoto Nagata, Hiromasa Inoue, Mitsuko Kondo, Takahiko Horiguchi, Nobuaki Miyahara, Nobuyuki Hizawa, Masayuki Hojo, Noboru Hattori, Naozumi Hashimoto, Akira Yamasaki, Toru Kadowaki, Tomoki Kimura, Mari Miki, Hirokazu Taniguchi, Mikio Toyoshima, Tetsuji Kawamura, Osamu Matsuno, Yoko Sato, Hironobu Sunadome, Tadao Nagasaki, Tsuyoshi Oguma, Toyohiro Hirai, on behalf of the BEXAS study.

**Additional Method**

**Details of Questionnaires**

1) Demographic data, (2) patterns of airway lesions, (3) symptoms and auscultation findings, (4) treatment and its evaluation by attending physicians, (5) frequencies of exacerbation, bronchopneumonia, and hospitalisation, (6) the latest and oldest laboratory data and pulmonary function, and (7) computed tomography findings, sputum culture, exhaled nitric oxide (FeNO), and percutaneous oxygen saturation at the latest and oldest or at the diagnosis of airway lesions were obtained.

1) Basic demographic data included the following: sex, age, height, weight, follow-up period, smoking history, occupational history, comorbidities (tuberculosis sequelae, COPD, diffuse pan-bronchiolitis, primary ciliary dyskinesia, neutrophilic chronic rhinosinusitis, eosinophilic chronic rhinosinusitis, pollinosis, rheumatoid arthritis, diabetes mellitus, gastroesophageal reflux disease, premature birth), age at asthma diagnosis, items for assuming asthma (repetitive symptoms of dyspnoea, wheeze, chest tightness or cough, reversible airflow limitation, airway hyperresponsiveness, FeNO > 35 ppb, sputum eosinophilia, atopic predisposition, blood eosinophil count > 300/μL) (1).

2) Morphological and inflammatory patterns of accompanying airway lesions, i.e., bronchiectasis (allergic bronchopulmonary mycosis, other eosinophilic, non-eosinophilic, unknown) and bronchiolitis (eosinophilic, non-eosinophilic, unknown), and timing of the diagnosis of airway lesions.

3) Symptoms and auscultation findings in stable and exacerbated phases. Auscultation findings included wheeze/rhonchi, squawk, and coarse crackles.

4) Current and previous use of inhaled corticosteroids, oral corticosteroids, long-acting β_2_ agonists, long-acting muscarinic antagonists, macrolides, antifungals, biologics, mucolytic agents, and respiratory physiotherapy/airway clearance. Overall, 0–250 μg/day of inhaled corticosteroids, equivalent to fluticasone propionate, was considered as zero to low dose, and ≥250 μg/day was considered as moderate to high dose. Attending physicians were asked for the most effective treatment in the long-term management and exacerbation phase.

5) Frequency of exacerbation requiring systemic corticosteroid and antibiotics in the last 2 years. Frequency of bronchopneumonia and hospitalisation in the last 5 years. The number of frequencies was given for each year, and ≥3 times were presented as 3.

6) The latest and oldest laboratory data included blood tests (white blood cells and their fractionation, serum C-reactive protein, serum IgE, and serum IgG) and pulmonary function.

7) Computed tomography (CT) findings, detection of pathogenic bacteria in the sputum (none, *Staphylococcus aureus*, *Streptococcus pneumonia*, *Haemophilus influenzae*, *Moraxella catarrhalis*, *Klebsiella pneumoniae*, *Serratia marcescens*, *Pseudomonas aeruginosa [P. aeruginosa]*, non-tuberculous mycobacteria, *Aspergillus*, and others), FeNO, and percutaneous oxygen saturation at the latest and oldest or at the diagnosis of airway lesions. The degree of airway enlargement was evaluated using the modified Reiff score (mReiff score) (2). Bronchiolitis was defined when centrilobular nodules or tree-in-bud signs were present in one or more lobes, and its severity was evaluated by counting the number of affected lobes. The lingula was defined as one lobe, and a total of six lobes were evaluated. A total of 162 patients (75%) were assessed by an attending physician and the first author (NN) of the manuscript. In these cases, NN evaluated the degree of airway dilation in a blinded manner. A total of 54 (25%) patents were assessed only by the attending physician due to ethical issues at the institution.

**Sample Size**

Our aim was to enrol approximately 200 patients with refractory asthma complicated by bronchiectasis. This number was estimated based on the fact that the number of patients with bronchiectasis in Japan was reported as 25,000 (3) and that the prevalence of asthma in patients with bronchiectasis in Japan was 7% (4). This survey—sent to 732 institutions—assumed a response rate of 10%.

**Statistical Analysis**

Analysis was performed using JMP version 15. Receiver operating characteristic curve analysis was performed to determine cut-off values of FeNO for bronchopneumonia and the detection of pathogenic bacteria in the sputum. Two or more groups were compared using χ^2^ test, Fisher’s exact test, Wilcoxon rank-sum test, and Kruskal–Wallis test, where deemed appropriate. Multiple comparison test was performed using the Steel–Dwass test. The Spearman correlation coefficient was used to analyse the relationships among data. Logistic multivariate analysis was performed to determine variables predictive of inflammatory group transition. No interaction terms were used. Wilcoxon signed-rank test was used to compare matched samples. When analysing frequencies of episodes, such as exacerbations in a defined period, cases that were followed up for at least the corresponding period were included. The numbers of responses for each variable are listed in Table and Figure legends. P < 0.05 was considered significant. Data are shown as means (SD).

**Additional Results**

**Impression of the Attending Physicians on Bronchiectasis/Bronchiolitis Complicated Asthma**

Most physicians were concerned about their patients’ repeated infectious episodes and chronic productive cough. For the treatment, the efficacy of ICS in the type 2-low group was considered lower than in the type 2-intermediate group and systemic corticosteroids were the most effective for exacerbations in all the three groups. Antibiotics were more frequently required and were more effective for exacerbations in the type 2-low group (Table S3).

**Differences Between the Stratified and Non-stratified Cases**

Overall, 142 cases were classified into the following three inflammatory groups: type 2-high (FeNO ≥ 32 ppb and blood eosinophil counts ≥ 320/μL; n = 34), type 2-intermediate (FeNO ≥ 32 ppb or blood eosinophil counts ≥ 320/μL; n = 40), and type 2-low (FeNO < 32 ppb and blood eosinophil counts < 320/μL; n = 68). The stratified 142 cases were younger and had shorter follow-up periods, more items related to asthma diagnosis, higher maximum dose of inhaled corticosteroid in the past, and higher forced expiratory volume in 1 second than non-stratified 74 cases (Table S1). The detection rate of *P. aeruginosa* in recent sputum in stratified cases tended to be lower than that in non-stratified cases. These suggest that stratified cases were more likely to have components of pure asthma than non-stratified cases.

**Stratification by Asthma Severity**

Severe asthma was defined when 1) patients were treated with high doses of ICS and one or more long-acting bronchodilators or 2) treated with regular OCS or biologics, or both. Total of 59% of the patients had severe asthma, and they had exacerbations requiring corticosteroids more frequently in the last two years than those with mild to moderate asthma [1.4 (1.2) vs 0.7 (1.0), p < 0.0001]. The recent FEV_1_/FVC was lower for severe asthma [65.0 (14.7) vs 69.2 (13.9), p = 0.04], while %FEV_1_ was comparable between the two groups [78.0 (31.1) vs 81.6 (26.1), p = 0.15]. There were no significant differences in mReiff scores [severe asthma vs non severe asthma; 3.2 (3.4) vs 3.3 (3.3), p = 0.75] and number of bronchiolitis-affected lobes on the latest CT [2.9 (2.4) vs 2.9 (2.2), p = 1.0] between the two groups.

**Analysis of Patients Not Receiving Anti-type 2 Biologics or Regular OCS (≥5 mg/day)**

Among the 142 stratified cases, 56 cases were administered with anti-type 2 biologics or regular OCS (≥5 mg/day). Excluding 56 cases from the analysis did not change the major characteristics of the type 2-high (n = 22), -intermediate (n = 24) and -low (n = 40) groups. The type 2-low group showed highest serum C-reactive protein levels (p = 0.02) among the three groups, and sputum culture of patients in type 2-low group revealed gram-negative bacteria (GNB) and *P. aeruginosa* most frequently among the three inflammatory groups. The presence of exacerbation requiring antibiotics and bronchopneumonia differed among the three groups (p = 0.009 and p = 0.02, respectively); however, the frequency of asthma exacerbation requiring systemic corticosteroids did not show differences among the groups. The transition pattern of inflammatory types in cases that were followed up with for ≥2 years (n = 51) is shown in Figure S1. All cases, except for one case in the former type 2-low group (n = 15), remained in the current type 2-low group. Among 36 cases of the former type 2-high or -intermediate groups, 9 (25%) transitioned to the current type 2-low group.

**FeNO Levels that Predicted the Presence of Bacteria in Sputum.**

FeNO levels that predicted the presence of GNB and *P. aeruginosa* in sputum were determined using ROC curve analysis. The area under the curves (AUCs) were 0.74 for GNB, with a sensitivity of 79% and specificity of 68% at FeNO 23 ppb, and 0.74 for *P. aeruginosa*, with a sensitivity of 68% and specificity of 73% at FeNO 17 ppb. The bacteria detection rates were significantly higher at lower FeNO values (Figure S2).

**Details of Airway Lesions and Their Transition Patterns**

Overall, the mean (SD) of the current mReiff score was 3.2 (3.4) (n = 201), and lobes affected by bronchiolitis was 3.0 (2.3) (n = 192). Mean of the former mReiff score was 2.6 (2.9) (n = 182), which was significantly lower than the current mReiff score (p < 0.0001, n = 174). Former lobes affected by bronchiolitis was 3.1 (2.2) (n = 172). The mean period between the latest CT data and former or at the diagnosis of airway lesions was 5.7 (4.4) years. The number of lobes affected by bronchiolitis on the latest CT was weakly associated with the forced expiratory volume in 1 second (% predicted) (rho = −0.22, p = 0.005, n = 167), the frequency of bronchopneumonia (rho = 0.31, p < 0.0001, n = 154), exacerbations requiring antibiotics (rho = 0.26, p = 0.001, n = 152), and exacerbations requiring systemic corticosteroid use (rho = 0.19, p = 0.02, n = 145) in the last two years.

The transition pattern of airway lesions on CT was analysed in patients, who were followed for ≥2 years (n = 116). Among 36 cases with bronchiolitis alone in the past, 11 (30.6%) currently presented with bronchiectasis (Figure S6). Lastly, Figure S5 shows the changes in mReiff scores and number of lobes affected by bronchiolitis stratified into high’-to-high’, high’-to-low, and low-to-low groups. In the high’-to-low group, the mReiff score significantly increased over time (former 2.8 vs current 3.9, p = 0.01).

**Supplementary Reference**

1. Hashimoto S, Sorimachi R, Jinnai T, Ichinose M. Asthma and chronic obstructive pulmonary disease overlap according to the Japanese Respiratory Society diagnostic criteria: The Prospective, Observational ACO Japan Cohort Study. Adv Ther 2021;38:1168-1184.

2. Chalmers JD, Goeminne P, Aliberti S, McDonnell MJ, Lonni S, Davidson J, et al. The bronchiectasis severity index. An international derivation and validation study. Am J Respir Crit Care Med 2014;189:576-585.

3. Data from Ministry of Health, Labor and Welfare. https://www.mhlw.go.jp/toukei/saikin/hw/kanja/05syoubyo/suiihyo31.html.

4. Kadowaki T, Yano S, Wakabayashi K, Kobayashi K, Ishikawa S, Kimura M, et al. An analysis of etiology, causal pathogens, imaging patterns, and treatment of Japanese patients with bronchiectasis. Respir Investig 2015;53:37-44.

**Supplementary Figure Legends**

**Figure S1.** Transition patterns of inflammatory types in cases who were not receiving anti-type 2 biologics or regular oral corticosteroids (≥5 mg/day) and were followed up for ≥2 years (n = 51). Red flow indicates former type 2-high group (n = 15), yellow flow indicates former type 2-intermediate group (n = 21) and blue flow indicates former type 2-low inflammatory group (n = 15).


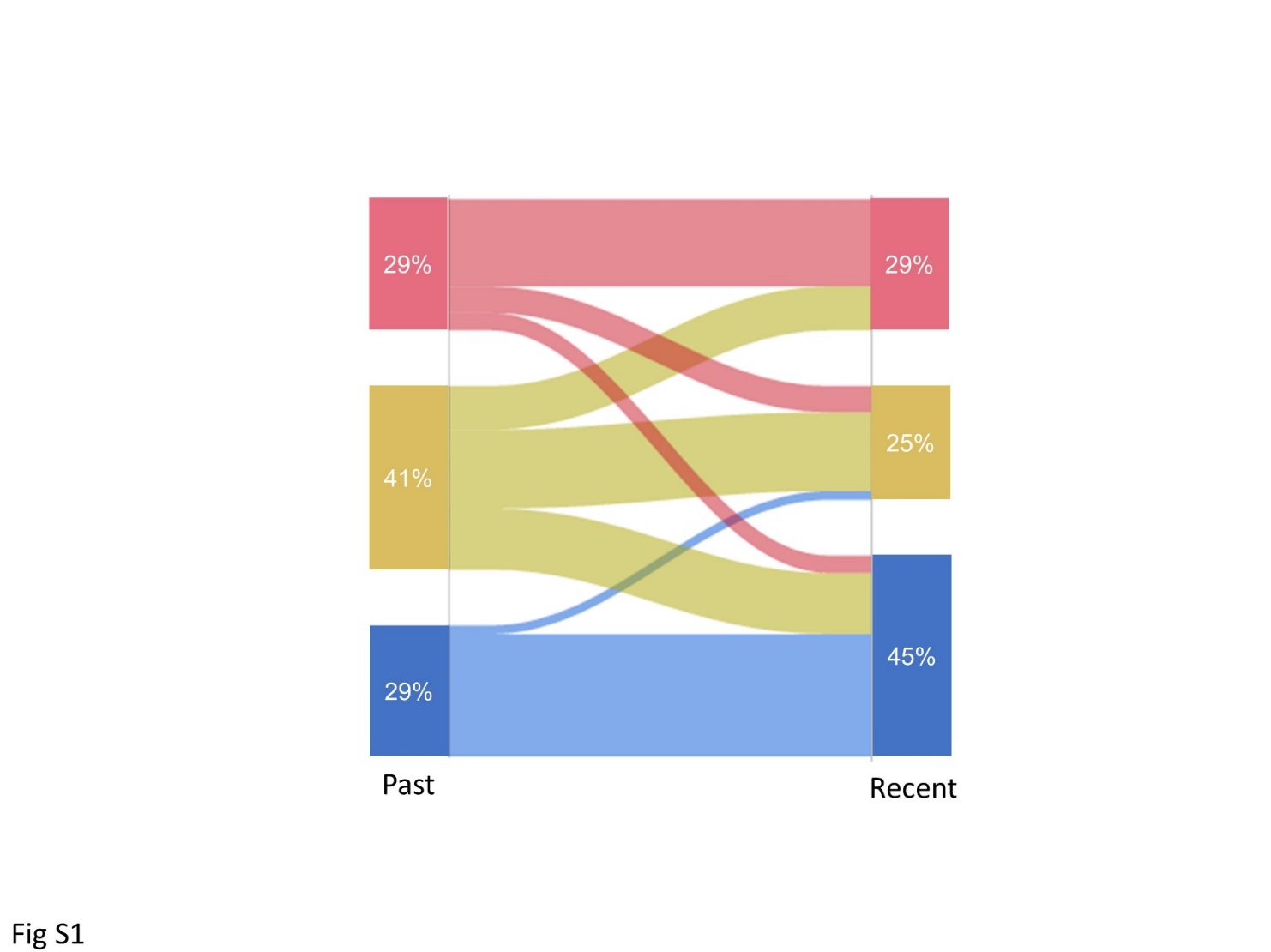


**Figure S2.** Receiver operating characteristic (ROC) curve for current levels of exhaled nitric oxide (FeNO) that reflected the presence of A) gram-negative bacteria (GNB) and B) *P. aeruginosa* in the sputum. Detection rate of C) GNB and D) *P. aeruginosa* in the sputum according to the FeNO levels, as determined by ROC curve analysis.


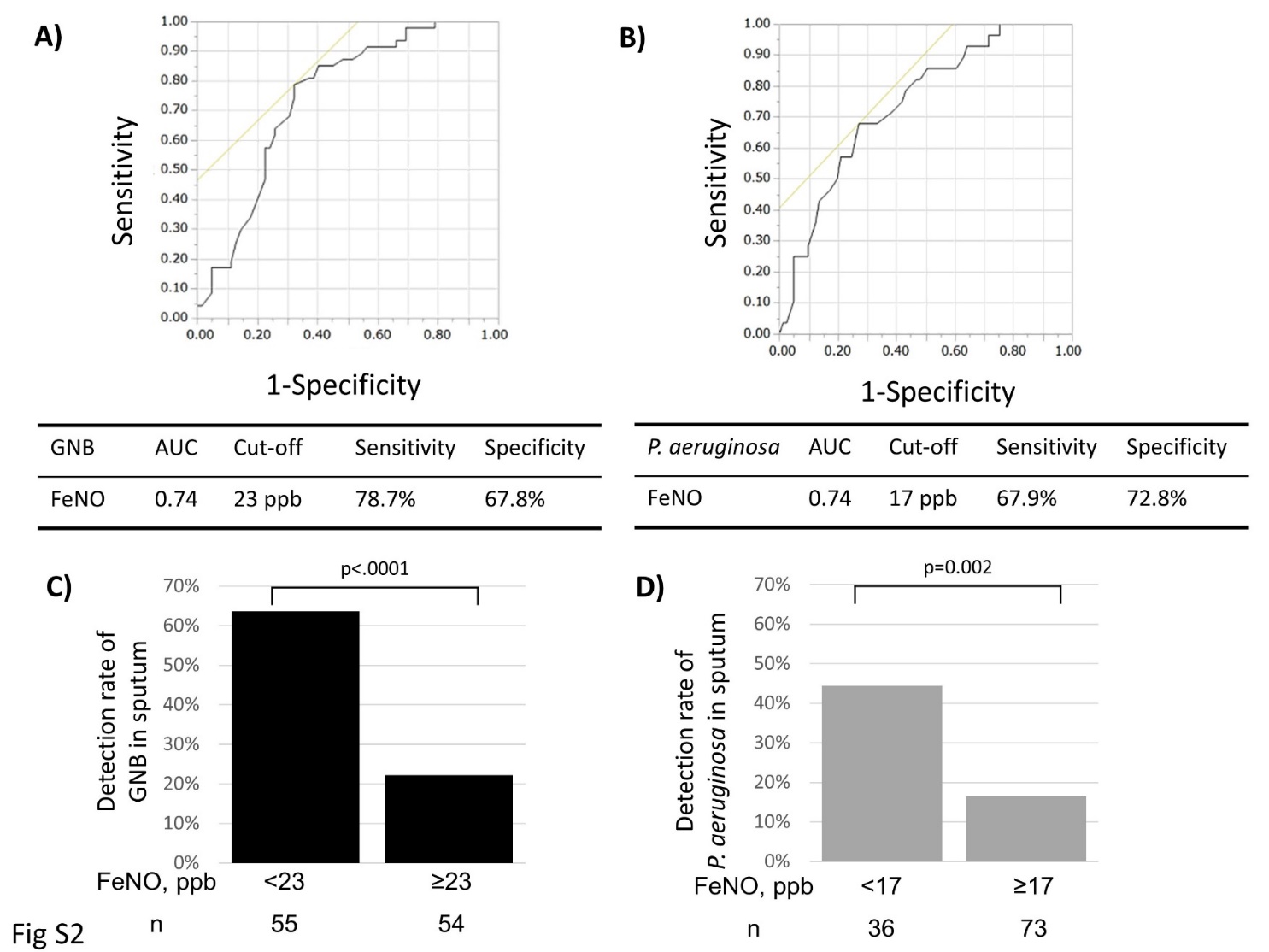


**Figure S3.** A) Modified Reiff score and B) number of lobes affected by bronchiolitis in low and high exhaled nitric oxide (FeNO) groups. Recent indices were analysed.

Boxes and bars indicate upper, lower, and median quartiles.


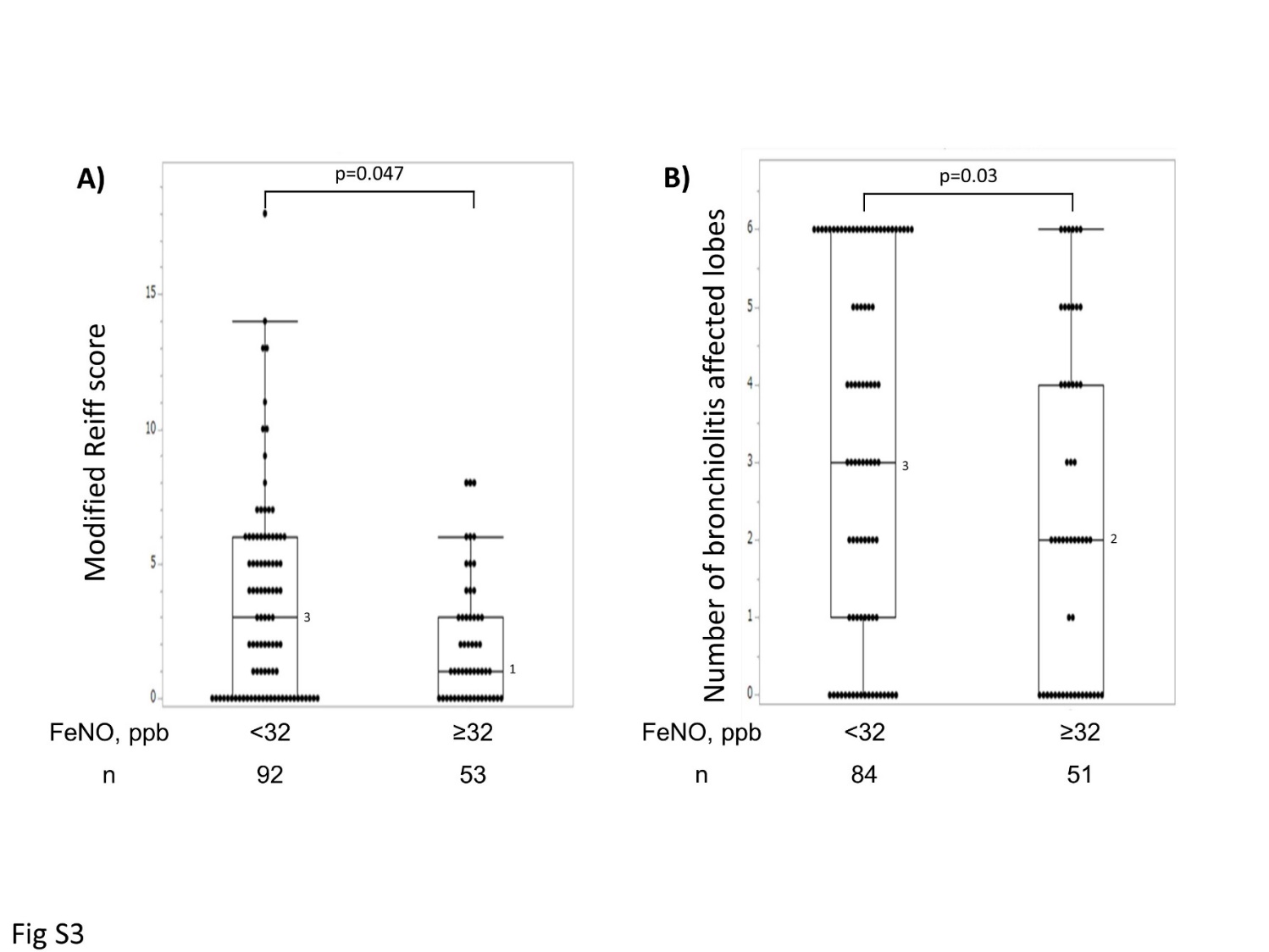


**Figure S4.** Patterns of cases with exacerbations requiring systemic corticosteroids (SCS) and antibiotics and bronchopneumonia, according to the transition patterns of inflammatory groups (p = 0.056 among the three groups). Red bar indicates cases with three types of episodes in the last 2 years, i.e., exacerbations requiring SCS and antibiotics, and bronchopneumonia; orange bar, exacerbations requiring SCS and antibiotics; yellow bar, exacerbations requiring SCS only: blue bar, bronchopneumonia and exacerbations requiring antibiotics; purple bar, bronchopneumonia only; green bar, exacerbations requiring antibiotics only. Complete answers were missing from two cases in the low-to-low group, four in the high’-to-low group, and eight in the high’-to-high’ group. Ratios of cases with exacerbation requiring antibiotics (p = 0.01) and bronchopneumonia (p = 0.006) were significantly different among the three groups.


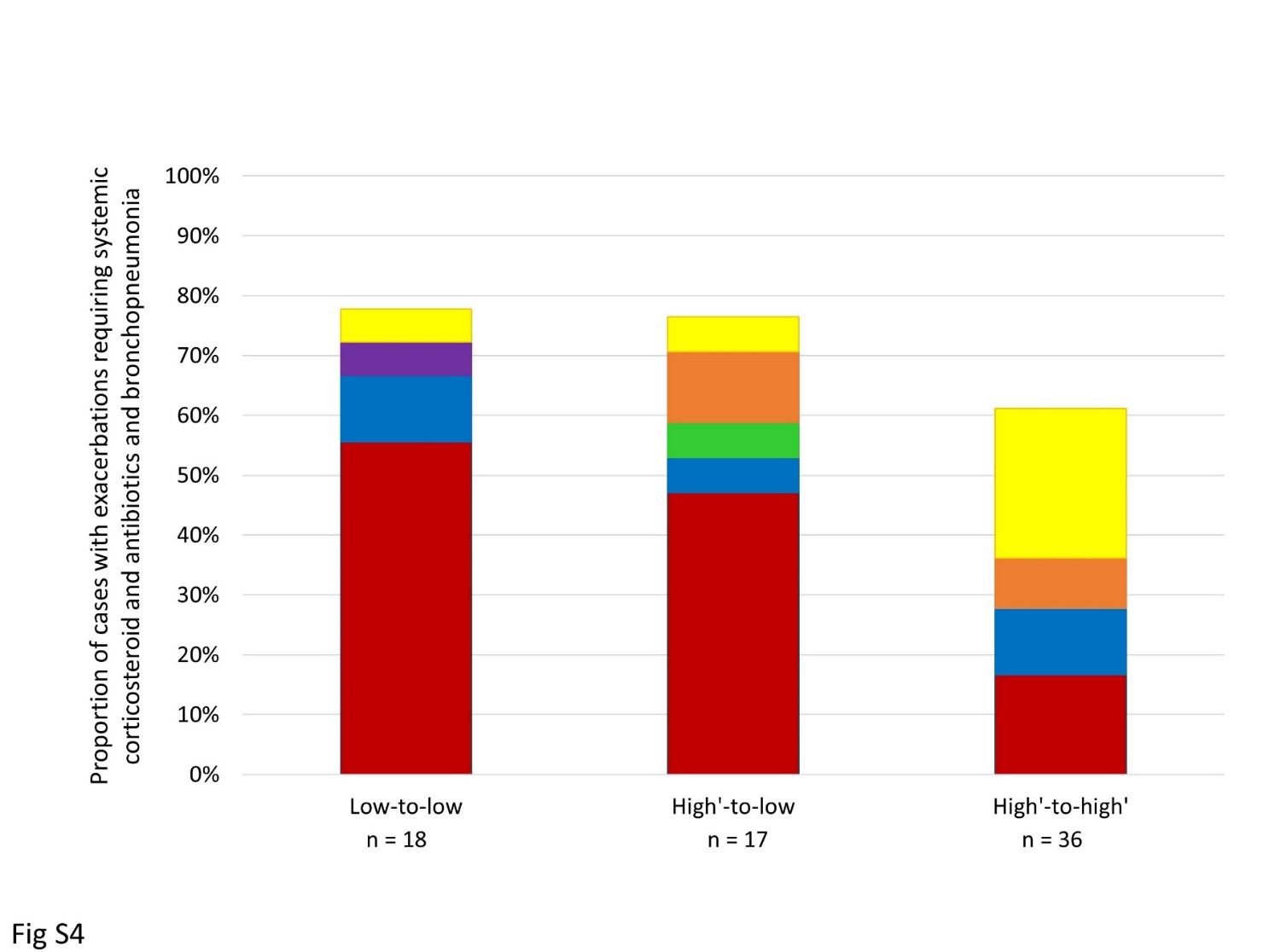


**Figure S5.** Changes in terms of A) inhaled corticosteroid (ICS) doses (equivalent to fluticasone propionate), B) modified Reiff scores, C) number of lobes affected by bronchiolitis, D) detection rates of gram-negative bacteria (GNB) in sputum and E) detection rates of *P. aeruginosa* in sputum over time. Blue line indicates low-to-low group, yellow line indicates high’-to-low group and pink line indicates high’-to-high’ group. The numbers of cases in the low-to-low, high’-to-low, and high’-to-high’ groups were 17/19/35 for A), 19/21/40 for B); 15/21/34 for C); 17/15/32 for D) and 17/15/32 for E).


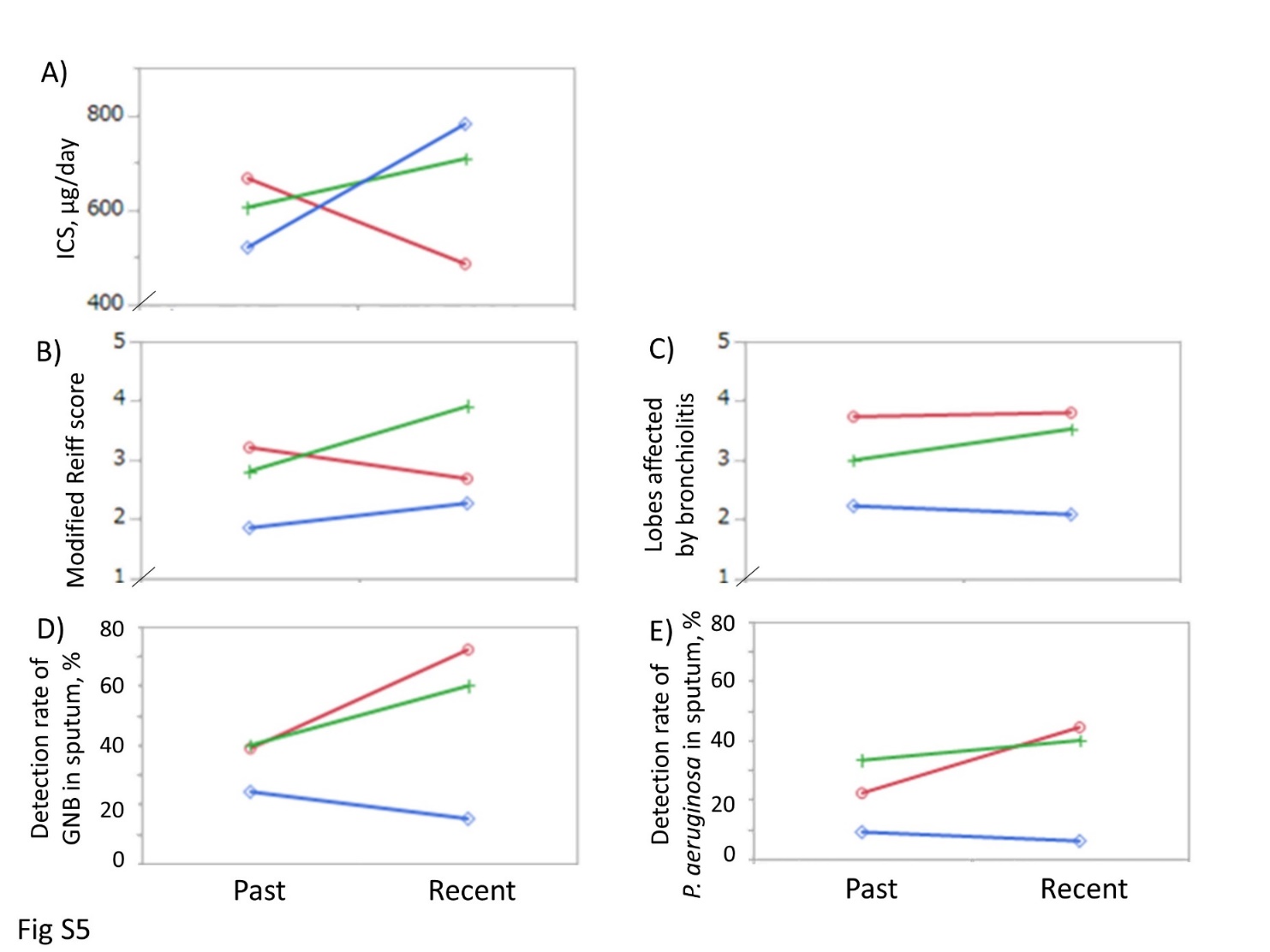


**Figure S6.** Transition patterns of airway lesions, i.e., bronchiectasis, bronchiolitis, and both, in cases followed for 2 years or more. A) In all cases regardless of type 2 inflammation level, purple flow indicates former bronchiectasis only (n = 17); orange flow, former bronchiolitis only (n = 36); green flow, bronchiectasis and bronchiolitis in the past (n = 63); B) current type 2-high group (n = 14), C) current type 2-intermediate group (n = 17) and D) current type 2-low group (n = 41).


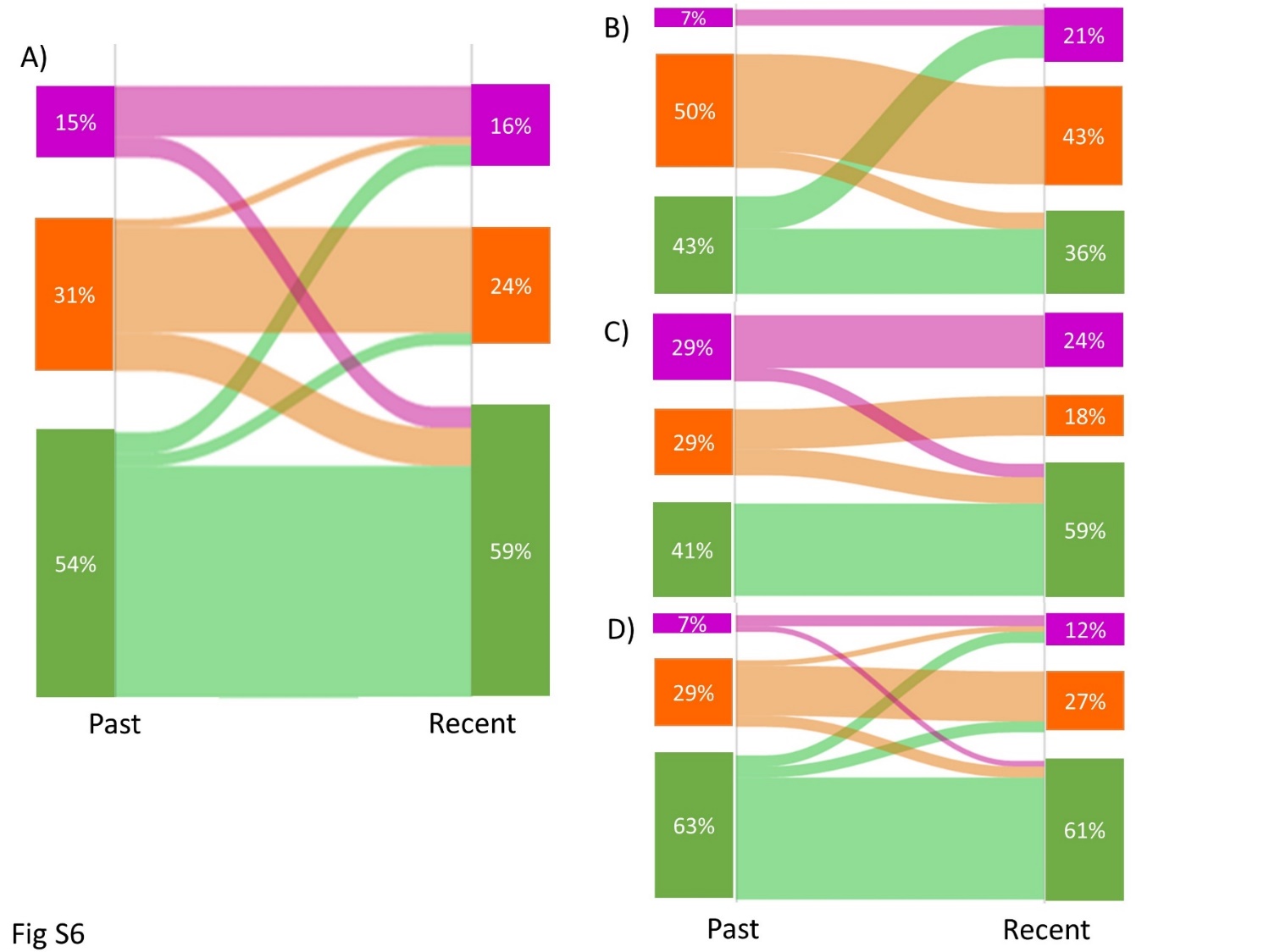


**Figure S7.** Trajectories of blood eosinophil counts of patients with ≥3 data points allocated to A) high’-to-high’, B) high’-to-low and C) low-to-low groups. Patients represented by grey and brown lines in A), who received oral corticosteroids or anti-type 2 biologics, or both, had elevated exhaled nitric oxide (>100 ppb), and they were allocated to the high’-to-high group.


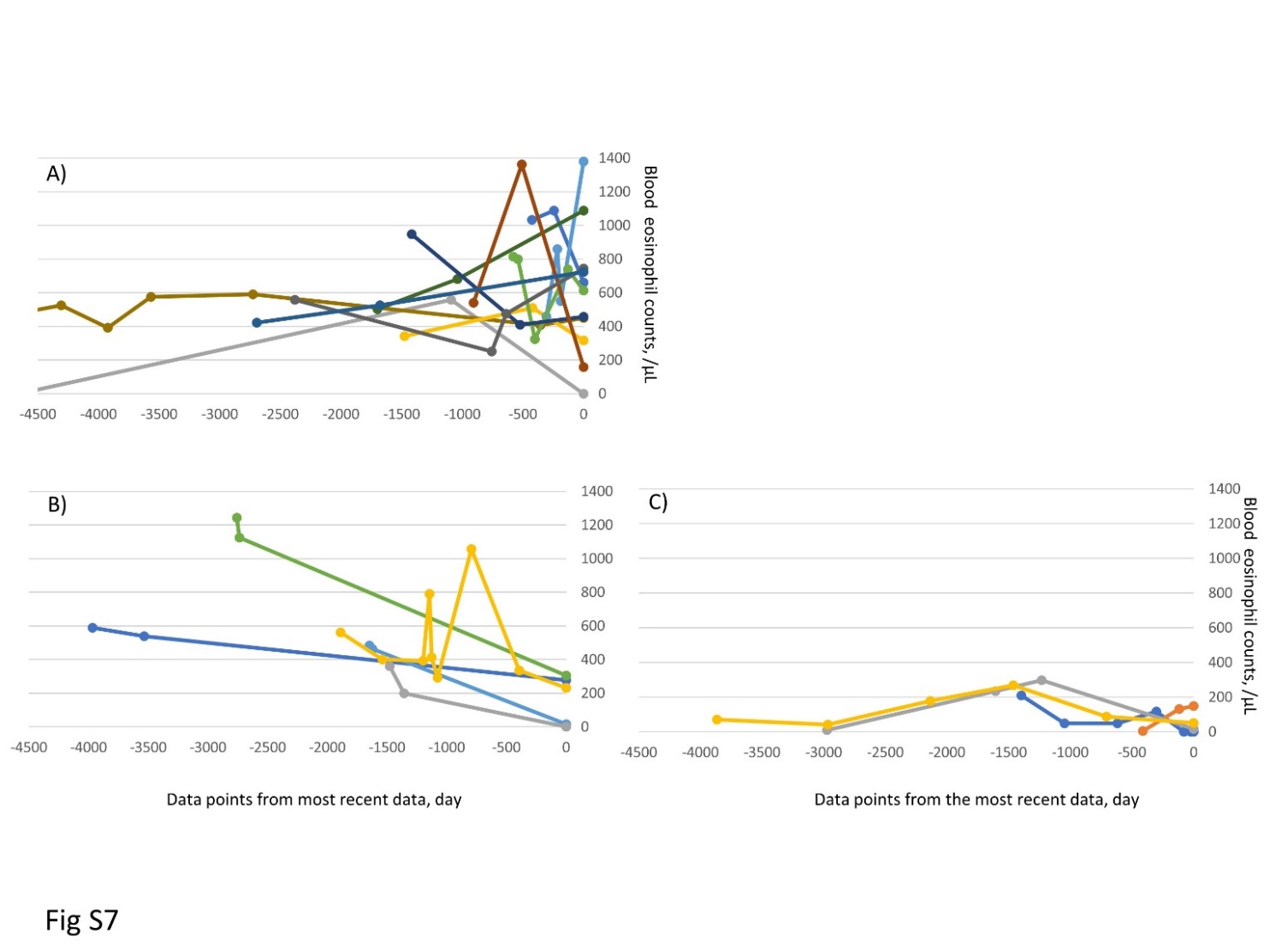


**Table S1.** Characteristics of patients who were and were not stratified.

|  | **Stratified cases**  **N = 142** | | **Non-stratified cases**  **N = 74** | | **p**  **-value** |
| --- | --- | --- | --- | --- | --- |
|  | **N^†^** |  | **N^†^** |  |  |
| Age, years | 142 | 63.2 ± 14.8 | 74 | 67.7 ± 14.0 | 0.02 |
| Sex, male, n (%) | 142 | 45 (31.7) | 74 | 31 (41.9) | 0.14 |
| Body mass index, kg/m^2^ | 121 | 22.8 ± 4.2 | 62 | 22.5 ± 3.7 | 0.77 |
| Follow-up period, years | 136 | 7.0 ± 5.8 | 71 | 9.8 ± 8.0 | 0.02 |
| Smoking history, never/past/current, % | 140 | 68/28/4 | 72 | 67/29/4 | 0.98 |
| Age at diagnosis of asthma, years | 135 | 42.5 ± 21.6 | 68 | 45.3 ± 21.5 | 0.36 |
| Number of items related to asthma diagnosis | 141 | 3.0 ± 1.4 | 71 | 2.2 ± 1.3 | <0.0001 |
| Age at diagnosis of bronchiectasis/bronchiolitis, years | 122 | 57.9 ± 16.6 | 63 | 59.8 ± 12.8 | 0.78 |
| Period from asthma diagnosis to bronchiectasis/bronchiolitis diagnosis, years | 117 | 14.9 ± 16.6 | 66 | 13.7 ± 20.3 | 0.42 |
| Current ICS dose, μg/day (eq. to FP) | 139 | 687 ± 367 | 66 | 631 ± 440 | 0.21 |
| Maximum dose of ICS in the past, μg/day (eq. to FP) | 139 | 509 ± 415 | 66 | 357 ± 406 | 0.01 |
| OCS use, never/past/current, n | 137 | 83/21/33 | 71 | 40/16/15 | 0.55 |
| Current data: |  |  |  |  |  |
| Blood eosinophil counts, /μL | 142 | 398 ± 563 | 56 | 330 ± 366 | 0.79 |
| Serum total IgE, IU/mL | 88 | 415 ± 550 | 39 | 1109 ± 2629 | 0.63 |
| Serum C-reactive protein, mg/dL | 116 | 1.1 ± 2.6 | 55 | 1.2 ± 2.2 | 0.86 |
| Exhaled nitric oxide, ppb | 142 | 41.6 ± 46.6 | 12 | 20.2 ± 10.5 | 0.12 |
| %FEV_1_, % | 126 | 83.5 ± 30.0 | 62 | 72.5 ± 23.9 | 0.009 |
| FEV_1_/FVC, % | 126 | 67.3 ± 14.5 | 62 | 66.1 ± 13.9 | 0.52 |
| Modified Reiff score | 134 | 3.0 ± 3.3 | 67 | 3.6 ± 3.5 | 0.27 |
| Number of lobes affected by bronchiolitis | 124 | 2.9 ± 2.3 | 68 | 3.0 ± 2.3 | 0.76 |
| Sputum *S. pneumonia* (+), n (%) | 102 | 12 (11.8) | 54 | 4 (7.4) | 0.39 |
| Gram-negative bacteria (+), n (%) | 102 | 43 (42.2) | 54 | 29 (53.7) | 0.17 |
| *P. aeruginosa* (+), n (%) | 102 | 26 (25.5) | 54 | 21 (38.9) | 0.08 |
| Presence of exacerbations requiring systemic corticosteroid in the last 2 years*, n (%) | 104 | 58 (55.8) | 57 | 29 (50.9) | 0.55 |
| Presence of exacerbations requiring antibiotics in the last 2 years*, n (%) | 110 | 51 (46.4) | 58 | 30 (51.7) | 0.51 |
| Presence of bronchopneumonia in the last 2 years*, n (%) | 112 | 46 (41.1) | 59 | 30 (50.9) | 0.22 |
| Presence of hospitalisation for exacerbation in the last 2 years*, n (%) | 112 | 22 (19.6) | 59 | 18 (30.5) | 0.11 |

Data are presented as means ± SD. FEV_1_: forced expiratory volume in 1 second, FVC: forced vital capacity, FP: fluticasone propionate, ICS: inhaled corticosteroid, OCS: oral corticosteroid. *Examined in cases that were followed for 2 years or more. ^†^Number of responses for each item.

**Table S2.** Features related to asthma and comorbidities according to the current inflammatory types

|  | **N^‡^** | **Type 2-low**  **N = 68** | **Type 2-intermediate**  **N = 40** | **Type 2-high**  **N = 34** | **p-value** |
| --- | --- | --- | --- | --- | --- |
| Number of items related to asthma diagnosis | 141 | 2.5 ± 1.4^§¶^ | 3.3 ± 1.0 | 3.9 ± 1.3 | <0.0001 |
| Number of subitems related to type 2 inflammation* | 136 | 0.8 ± 0.9^§¶^ | 1.5 ± 1.0 | 2.0 ± 0.8 | <0.0001 |
| Repetitive symptoms | 140 | 64 (95.5) | 38 (97.4) | 33 (97.1) | 0.85 |
| Reversible airflow limitation | 136 | 19 (29.2) | 9 (23.7) | 11 (33.3) | 0.66 |
| Airway hyperresponsiveness | 133 | 4 (6.2) | 3 (8.3) | 3 (9.4) | 0.83 |
| Exhaled nitric oxide> 35 ppb* | 138 | 17 (25.8) ^§¶^ | 25 (64.1) ^§^ | 31 (93.9) | <0.0001 |
| Sputum eosinophilia* | 137 | 14 (21.2) | 12 (31.6) | 11 (33.3) | 0.33 |
| Atopic predisposition* | 139 | 26 (38.8) | 21 (53.9) | 18 (54.6) | 0.19 |
| Blood eosinophil counts>300/μL* | 140 | 26 (38.2) ^§^ | 21 (53.9) | 24 (72.7) | 0.005 |
| Comorbidities |  |  |  |  |  |
| Tuberculosis sequelae | 132 | 2 (3.0) | 1 (2.8) | 0 (0) | 0.63 |
| COPD | 132 | 4 (6.1) | 1 (2.9) | 3(9.7) | 0.51 |
| Diffuse pan-bronchiolitis | 132 | 11 (16.7) | 3 (8.6) | 3(9.7) | 0.43 |
| Primary ciliary disorder | 132 | 1 (1.5) | 0 (0) | 0 (0) | 0.60 |
| Neutrophilic chronic rhinosinusitis | 132 | 22 (33.3) | 10 (28.6) | 11 (35.5) | 0.82 |
| Eosinophilic chronic rhinosinusitis | 133 | 7 (10.6) | 8 (22.2) | 8 (25.8) | 0.12 |
| Pollinosis | 132 | 10 (15.2) | 6 (17.1) | 9 (29.0) | 0.25 |
| Rheumatoid arthritis | 132 | 7 (10.6) | 2 (5.7) | 1 (3.2) | 0.39 |
| Diabetes mellitus | 133 | 5 (7.6) | 2 (5.6) | 3 (9.7) | 0.82 |
| Gastroesophageal reflux disease | 132 | 6 (9.1) | 0 (0) | 1 (3.2) | 0.13 |
| Premature birth | 131 | 0 | 0 | 0 | - |
| Auscultation in stable phase:  Wheeze/rhonchi | 122 | 17 (27.9) | 6 (18.2) | 3 (10.7) | 0.16 |
| Squawk | 122 | 4 (6.6) | 0 (0) | 1 (3.6) | 0.31 |
| Coarse crackles | 122 | 12 (19.7) | 1 (3.0) | 1 (3.6) | 0.02 |
| Auscultation in exacerbated phase: Wheeze/rhonchi | 136 | 56 (83.6) | 30 (79.0) | 26 (83.9) | 0.81 |
| Squawk | 135 | 6 (9.1) | 5 (13.2) | 3 (9.7) | 0.80 |
| Coarse crackles | 135 | 23 (34.9) | 6 (15.8) | 4 (12.9) | 0.02 |
| Inflammatory types^†^:  eosinophilic/non-eosinophilic/unknown, n (%) | 128 | 7/31/25 (11/49/40) | 12/8/16  (33/22/44) | 5/5/19  (17/17/66) | 0.002 |

Data are presented as means ± SD and number (% of responded cases), ^†^Assigned by attending physicians, ^‡^Number of responses for each item, ^§^p < 0.05 vs type 2-high group, ^¶^ p < 0.05 vs type 2-intermediate group.

**Table S3.** Most effective treatment evaluated by attending physicians

|  | **N*** | **Type 2-**  **low**  **N = 68** | **Type 2-intermediate**  **N = 40** | **Type 2-high**  **N = 34** | **p-value** |
| --- | --- | --- | --- | --- | --- |
| Most effective treatment in stable phase |  |  |  |  |  |
| Inhaled corticosteroid | 140 | 16 (24.2) ^†^ | 21 (52.5) | 16 (47.1) | 0.007 |
| Oral corticosteroid | 142 | 13 (19.1) | 5 (12.5) | 7 (20.6) | 0.60 |
| Long-acting β_2_ agonist | 142 | 4 (5.9) | 5 (12.5) | 4 (11.8) | 0.43 |
| Long-acting muscarinic antagonist | 142 | 7 (10.3) | 0 (0) | 3 (8.8) | 0.12 |
| Macrolide therapy | 142 | 15 (22.1) | 5 (12.5) | 3 (8.8) | 0.18 |
| Biologics | 142 | 11 (16.2) | 8 (20.0) | 4 (11.8) | 0.63 |
| Physiotherapy or airway clearance | 142 | 1 (1.5) | 2 (5.0) | 0 (0) | 0.29 |
| Most effective treatment during exacerbations |  |  |  |  |  |
| Systemic corticosteroid | 136 | 30 (45.5) | 18 (47.4) | 19 (59.4) | 0.42 |
| Short-acting β_2_ agonist | 130 | 3 (4.7) | 1 (2.9) | 1 (3.2) | 0.88 |
| Antibiotics | 131 | 18 (27.7) ^†‡^ | 2 (5.7) | 0 (0) | 0.0004 |

Numbers (% of responded cases) are presented. *Number of responses for each item, ^†^p < 0.05 vs type 2-intermediate group, ^‡^ p < 0.05 vs type 2-high, ^‡^

**Table S4**. Features related to asthma according to the transition patterns of inflammatory types

|  | N^‡^ | Low-to-low  N = 20 | High’-to-low  N = 21 | High’-to-high’  N = 44 | p-value |
| --- | --- | --- | --- | --- | --- |
| Number of items related to asthma diagnosis | 85 | 2.0 ± 1.1^§¶^ | 3.4 ± 1.4 | 3.8 ± 1.1 | <0.0001 |
| Number of subitems related to type 2 inflammation* | 83 | 0.6 ± 0.8^§¶^ | 1.4 ± 1.0 | 2.0 ± 0.9 | <0.0001 |
| Repetitive symptoms, n (%) | 85 | 19 (95.0) | 21 (100) | 43 (97.7) | 0.57 |
| Reversible airflow limitation, n (%) | 83 | 5 (26.3) | 8 (38.1) | 11 (25.6) | 0.56 |
| Airway hyperresponsiveness, n (%) | 83 | 0 (0) | 3 (14.3) | 5 (11.6) | 0.25 |
| Exhaled nitric oxide> 35 ppb*, n (%) | 84 | 0 (0) ^§¶^ | 12 (57.1) ^¶^ | 35 (81.4) | <0.0001 |
| Sputum eosinophilia*, n (%) | 83 | 5 (26.3) | 5 (23.8) | 17 (39.5) | 0.36 |
| Atopic predisposition*, n (%) | 84 | 5 (25.0) | 11 (52.4) | 23 (53.5) | 0.09 |
| Blood eosinophil counts>300/μL*, n (%) | 84 | 6 (30.0) ^¶^ | 12 (57.1) | 32 (74.4) | 0.004 |
| Inflammatory types^†^:  eosinophilic/non-eosinophilic/unknown, n (%) | 76 | 0/12/6  (0/67/33) | 6/8/6  (30/40/30) | 8/10/20  (21/26/53) | 0.02 |

Data are presented as means ± SD. ^†^Assigned by attending physicians. ^‡^Number of responses for each item, ^§^p < 0.05 vs High’-to-low, ^¶^p < 0.05 vs High’-to-high’ group.
